# Antithrombotic regimens for the prevention of major adverse cardiac events in chronic coronary syndrome: A systematic review and network meta-analysis

**DOI:** 10.3389/fcvm.2023.1040936

**Published:** 2023-04-06

**Authors:** Gustavo Lenci Marques, Arthur Mendonça Albuquerque, Gabriela Romaniello, Fernanda Proença Lepca Bozzi, Gustavo Pereira da Cunha, Gabriel Savogin Andraus, Gabriel Hastreiter, Barbara Maniesi, Cristina Baena, Murilo Guedes

**Affiliations:** ^1^ Postgraduate Program in Health Sciences, School of Medicine, Pontificia Universidade Catolica do Parana, Curitiba, Brazil; ^2^ Department of Internal Medicine, Universidade Federal do Parana, Curitiba, Brazil; ^3^ School of Medicine, Universidade Federal do Rio de Janeiro, Rio Janeiro, Brazil; ^4^ Department of Cardiology, Hospital Universitário Cajuru, Curitiba, Brazil; ^5^ Department of Cardiology, Hospital Marcelino Champagnat, Curitiba, Brazil; ^6^ School of Medicine, Pontifícia Universidade Católica do Paraná, Curitiba, Brazil

**Keywords:** coronary arter disease, major adverse cardiac event (MACE), platelet aggregation inhibitors, anticoagulants, secondary prevention

## Abstract

**Backgroud:**

Antithrombotic therapy is the cornerstone of chronic coronary syndrome (CCS) management. However, the best treatment option that optimally balances bleeding risk and efficacy remains undefined. Our objective was to evaluate the effectiveness and safety of antithrombotic options and identify the optimal treatment option for patients with CCS.

**Methods:**

We used the MEDLINE, CENTRAL and Embase databases to search for randomized controlled trials with follow-up periods longer than 12 months that compared aspirin (ASA) monotherapy with other antithrombotic therapies in patients with CCS. The Preferred Reporting Items for Systematic Reviews and Meta-Analyses guidelines were used. Extracted data [hazard ratios (HR)] were pooled using Bayesian fixed-effect models, allowing the estimation of credible intervals (CrI) and posterior probabilities of benefit, harm, and practical equivalence. Confidence in the results was assessed with the Confidence In Network Meta-Analysis (CINeMA) tool. The primary efficacy and safety outcomes were major adverse cardiovascular events (MACE) and primary bleeding, respectively. Secondary outcomes were acute myocardial infarction, ischemic stroke, all-cause, and cardiovascular-specific mortality.

**Results:**

Five trials with a total of 80,605 patients were included. Mean patient age ranged from 61 to 69 years, while 20.3% to 31.4% were women. The reference treatment was ASA monotherapy. ASA + prasugrel 10 mg and clopidogrel 75 mg monotherapy presented the greatest benefit for MACE [HR 0.52 (95% CrI, 0.39–0.71); and 0.68 (95% CrI, 0.54–0.88)]. There was a probability of 98.8% that ASA + ticagrelor was practically equivalent to ASA monotherapy. Regarding the primary bleeding outcome, clopidogrel 75 mg monotherapy performed best [HR 0.64 (0.42, 0.99)]. There was a probability of 97.4% that ASA + Prasugrel 10 mg increases bleeding (HR > 1.0). Secondary outcome results followed a similar treatment ranking pattern as in primary outcomes. Overall, CINeMA confidence ratings were judged as either low or very low.

**Conclusions:**

These results revealed that clopidogrel monotherapy might provide the best risk-benefit balance in treating CCS. However, low CINeMA confidence ratings may preclude more forceful conclusions. Our analysis suggests that current guidelines recommending ASA as first-line therapy for CCS management need to be revised to include additional pharmacological options.

## 1. Introduction

Chronic coronary syndrome (CCS) is characterized by the accumulation of obstructive or non-obstructive coronary atherosclerotic plaques, resulting in myocardial ischemia (1, 2). Recent guidelines on CCS treatment recommend low-dose aspirin (ASA) for long-term antithrombotic therapy (2, 3). However, in recent years, new evidence suggests that alternative antithrombotic therapies may provide better efficacy compared with ASA monotherapy in patients with CCS (4–8).

Multiple randomized controlled trials (RCTs) have tested the standard of care of ASA monotherapy compared with PY12 inhibitors, either in monotherapy or dual antiplatelet therapy, or direct oral anticoagulants (DOAC) therapy among patients with CCS (4–8). These studies suggest that alternative therapies might be effective in reducing major adverse cardiovascular outcomes (MACE), although with increased bleeding events (4–8).

While recent guidelines recommend ASA as the first-line therapy, they also recommend considering the use of an additional antithrombotic drug in patients with high/moderate ischemic risk (2). There are no studies that compare ASA + PY12 inhibitors with ASA + DOACs. Therefore, current recommendations are based on indirect comparisons between trials.

A 2021 network meta-analysis that evaluated antithrombotic agents in patients with CCS (4, 9) concluded that ASA + rivaroxaban was possibly the most favorable regimen in patients with CCS, while ASA + ticagrelor or rivaroxaban monotherapy yielded the worst risk-benefit (9). However, this meta-analysis excluded patients in the ASA + prasugrel arm in the DAPT study (4, 9), which may have influenced the results. Additionally, a new RCT comparing ASA monotherapy with clopidogrel was published in 2021 (7). An updated analysis of available RCTs on antithrombotic therapy among patients with CCS is warranted. Therefore, we performed a network meta-analysis of RCTs to evaluate the effectiveness and safety of antithrombotic agents in patients with CCS.

## 2. Methods

This systematic review and meta-analysis study was registered in the International Prospective Register of Systematic Reviews (PROSPERO; http://www. crd. york. ac. uk/prospero/) as CRD42022308499. This study was conducted according to the PRISMA statement for reporting systematic reviews and network meta-analyses (10).

### 2.1. Search strategy

A systematic search was performed of published medical research for RCTs that compared antithrombotic strategies for secondary prevention in patients with CCS. We searched the Cochrane Library, Embase, and MEDLINE from inception till May 2022, without language restrictions. The complete search strategy is provided in the Supplementary Material.

### 2.2. Inclusion criteria

Studies were included if they (1) were RCTs, (2) included non-pregnant patients >18 years with ASA monotherapy or other antithrombotic strategies, (3) were conducted in patients with CCS, and (4) were published in peer-reviewed journals. CCS was defined as a history of MI >12 months or coronary revascularization >6 months prior to enrollment or the presence of obstructive (≥50%) coronary plaques documented by catheterization or CTA (11). Previous trials comparing ASA doses demonstrated similar results; therefore, all dose regimens were clustered and included as ASA monotherapy (12). Studies reporting patients with atrial fibrillation were excluded because current guidelines recommend anticoagulation therapy in this population (13). Conference abstracts and presentations were also excluded because their results may not be conclusive and such publications undergo limited peer reviews. Open-label and blinded studies were included to avoid the exclusion of important RCTs. Finally, to assess the long-term efficacy and safety of antithrombotic therapies, only RCTs with follow-up durations >1 year were included in the review and analysis.

### 2.3. Outcome measures

The primary efficacy outcome was trial-defined MACE, while the primary safety outcome was trial-defined primary bleeding (hereafter referred to as bleeding outcome; see Supplementary Table S1 for each trial definition). The secondary outcomes were (1) acute myocardial infarction, (2) ischemic stroke, (3) all-cause mortality, and (4) cardiovascular-specific mortality.

We did not analyze each component of the trial-defined primary bleeding outcome because of the high heterogeneity across trials.

### 2.4. Data extraction

Two reviewers (GC and GH) independently reviewed the titles and abstracts of the articles returned from our initial search and determined their eligibility for inclusion. Discrepancies in eligibility decisions were addressed by a third reviewer (MG). Full-length articles were independently reviewed by two reviewers (GA and BM). Discrepancies in eligibility decisions were sent to a third reviewer (MG).

Two reviewers (GA and GH) extracted data from the RCTs. Data extracted from each RCT included patient, study-level characteristics and outcomes, extracted data included average age, median follow-up time, sex distribution, proportion of patients with a history of MI and stroke, and proportion of patients with relevant comorbidities at baseline.

A quality assessment of selected trials was conducted using the Cochrane Risk of Bias 2 (RoB 2) tool (14).

### 2.5. Statistical analysis

We conducted univariate network meta-analyses (NMAs) to estimate both direct and indirect estimates between treatments (one network per outcome) (15). We assumed that patients included in each trial were equally likely to be randomized to any treatment (transitivity), assessed by comparing the distributions of relevant clinical variables, such as the proportion of female patients, hypertension, diabetes, smoking, history of PCI or CAGB, and multivessel coronary disease (Supplementary Figure S1). NMAs also require the assumption that direct and indirect estimates are consistent (consistency). However, all networks presented in this article are “star-shaped” (16). That is, there are no direct and indirect estimates for the same treatment comparison; thus, we did not assess consistency.

We present results of fixed-effect NMAs, which allow inferences exclusively conditioned to the studies included in each network. We initially planned to fit random-effects NMAs (17); however, the analyses yielded implausibly wide parameters due to the assumption of an untenable common between-study heterogeneity (data not shown) (18).

The estimate of interest was the hazard ratio (HR), which was extracted from each RCT, converted to the log scale, and aggregated in NMAs with the normal likelihood. We accounted for within-trial correlation (19) because multi-arm trials were included in our analysis [Equations 7 and 9 from (20)]. Notably, one trial (5) reported separate HRs for ASA + ticagrelor 60 mg and ASA + ticagrelor 90 mg in the trial-defined primary bleeding outcome. To include these results in the “ASA + pooled ticagrelor” networks, we first fitted a frequentist fixed-effect pairwise meta-analysis including these two HRs only to estimate the ASA + pooled ticagrelor HR for the trial-defined primary bleeding outcome in the PEGASUS study.

To address dose inconsistencies in selected RCTs, we performed two sets of analyses: (1) we pooled results from all ticagrelor doses (generating a treatment referred to as “ASA + pooled ticagrelor”) and (2) we separated ticagrelor doses into 60 and 90 mg and analyzed their results. We considered NMAs that included ASA + pooled ticagrelor as our primary analyses. The THEMIS study initially randomized patients to ASA + Ticagrelor 90 mg but then switched the dose to 60 mg through a protocol amendment. The authors did not report results on patients that only used ASA + Ticagrelor 90 mg. Hence we only included THEMIS' results in “ASA + Pooled Ticagrelor” and “ASA + Ticagrelor 60 mg” network nodes.

We applied a Bayesian statistical framework (15), which updates “priors” (existing knowledge on the topic) with current data to generate a posterior distribution. These Bayesian fixed-effect meta-analyses has only one main parameter (average effect), for which we assigned a weakly informative prior [Normal (0, 1.5)]. To summarize marginal posterior distributions, we used medians and 95% highest-density intervals [hereafter, credible intervals (CrI)], defined as the narrowest interval containing 95% of the probability density function (21).

The Bayesian framework allows the estimation of clinically actionable probabilities (posterior probabilities). Hence, we estimated posterior probabilities for each treatment comparison (HR < 1.0, HR < 0.8, HR > 1.0, and HR > 1.25). Subsequently, we calculated the posterior probability of the treatments being practically equivalent, that is, HR between 0.8 and 1.25, represented by the region of practical equivalence (ROPE).

Using network plots, we assessed the network geometry and summarized the main results using forest plots (using ASA as the control group because all RCTs investigated this treatment). Each forest plot depicts the overall HRs with 95% CrI and accompanying posterior probabilities. Furthermore, we presented league tables to depict comparisons among all treatments (HRs and posterior probabilities). Thereafter, we estimated the ranking probabilities and surface under the cumulative ranking curves (SUCRA). Larger SUCRA values indicate better performance (22). Finally, ranking probabilities (median and 95% quantile intervals) were calculated to define the probability that each treatment was the best in each network (23).

The NMAs were fitted using Stan in the R package, multinma (23, 24). Four Markov chains were implemented with an initial warm-up phase of 2,000 iterations, followed by 4,000 iterations. We confirmed the convergence and adequate sampling of the models by checking trace plots, elevated effective sample sizes, and Rhats <1.01.

We assessed confidence in the results of our NMAs using CINeMA (25), which considers six domains: within-study bias, reporting bias, indirectness, imprecision, heterogeneity, and incoherence. The range of equivalence considered during the assessment of imprecision was between HR 0.8 and 1.25. Lastly, we summarized judgments across domains, referred to as “overall judgement”.

All analyses were conducted in R, version 4.1.2 (R Environment). The data and code are available at https://github.com/arthur-albuquerque/ARICOCAD_nma.

## 3. Results

**Figure 1:**
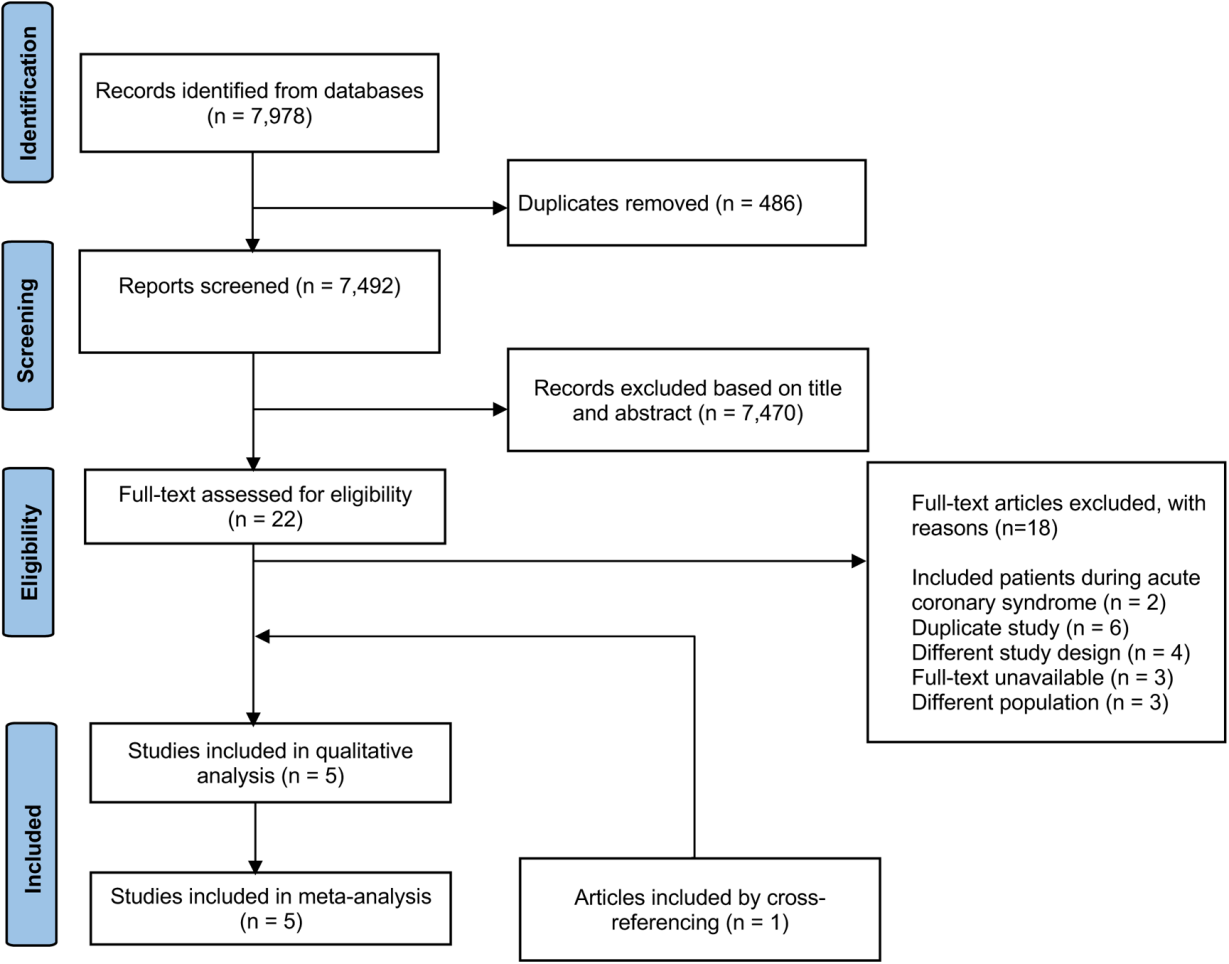
PRISMA flowchart of articles included in network meta-analysis. From 7,492 unique articles, we screened titles and abstracts and excluded 7,470 articles that did not meet inclusion criteria. The full-text of eligible articles (*n* = 22), including an article included by cross-referencing, was reviewed using inclusion and exclusion criteria to identify articles for data extraction (*n* = 5). All stages were conducted independently by 2 investigators.

The search strategy identified 7,978 citations, including 7,492 unique reports. Twenty-two full-text articles were retrieved after excluding 7,470 reports (Figure 1). Five RCTs comprising 80,605 patients were included in the review and meta-analyses. Seven different antithrombotic regimens were identified: ASA, ASA + prasugrel 10 mg, ASA + rivoroxaban 2.5 mg, ASA + clopidogrel 75 mg, clopidogrel 75 mg, ASA + ticagrelor, and rivaroxaban 5 mg.

Table 1 shows the baseline patient characteristics for each RCT. The mean age of patients was 61–69 years, and 20.3% to 31.4% were female patients. Comorbidities such as hypertension, dyslipidemia, and diabetes were common and well balanced between the trials and treatment arms (Supplementary Figure S1).

**Table 1:** Population baseline and trial characteristics.

Trial name	Initiation	Completion	Design	Analysis type	Follow-up (months)	Treatment arms	N (randomization)	Age^a^	Female	Current smoker	Hypertension	Diabetes	Prior CABG	Prior PCI	Multivessel disease	MACE outcome	Bleeding outcome
THEMIS	February 2014	May 2016	Double-blinded, multicenter, placebo-controlled trial	Modified intention-to-treat^b^	39.9	ASA + Ticagrelor	9,619	66 (61–72)	3,043 (31.6%)	1,056 (11.0%)	8,909 (92.6%)	9,619 (100%)	2,120 (22%)	5,558 (57.8%)	5,951 (61.9%)	736 (7,7%)	206 (2,1%)
						ASA	9,601	66 (61–72)	2,988 (31.1%)	1,038 (10.8%)	8,867 (92.4%)	9,601 (100%)	2,071 (21.6%)	5,596 (58.3%)	5,984 (62.3%)	818 (8,5%)	100 (1,0%)
COMPASS	March 2013	May 2016	Double-blinded, multicenter, placebo-controlled trial	Intention-to-treat	23.4	ASA	8261	69 (65–73)	1,646 (20%)	1,687 (20%)	6,218 (75%)	3,040 (37%)	2,586 (31%)	4,905 (59%)	5,043 (61%)	460 (5,6%)	105 (1,3%)
						Rivoroxaban 5 mg	8250	69 (65–73)	1,650 (20%)	1,680 (20%)	6,214 (75%)	3,015 (37%)	2,555 (31%)	4,986 (60%)	5,174 (63%)	411 (5,0%)	186 (2,2%)
						ASA + Rivoroxaban 2,5 mg	8313	69 (65–73)	1,736 (21%)	1,679 (20%)	6,280 (76%)	3,043 (37%)	2,074 (33%)	4,971 (60%)	5,252 (63%)	347 (4,2%)	164 (2%)
HOST-EXAM	March 2014	May 2018	Open-label, multicenter, placebo-controlled trial	Intention-to-treat	24	Clopidogrel 75 mg	2710	63,5 (10.7)	695 (25.6%)	545 (20.1%)	1,664 (61.4%)	925 (34.1%)	–	2,710 (100%)	855 (31.5%)	99 (3,7%)	33 (1,2%)
						ASA	2728	63,4 (10.7)	689 (25.3%)	581 (21.3%)	1,674 (61.4%)	935 (34.3%)	–	2,728 (100%)	844 (30.9%)	146 (5,5%)	53 (2,0%)
PEGASUS	October 2010	May 2013	Double-blinded, multicenter, placebo-controlled trial	Intention-to-treat	33	ASA + Ticagrelor 60 mg	7045	65.2 (8.4)	1,661 (23.6%)	1,206 (17.1%)	5,461 (77.5%)	2,308 (32.8%)	–	5,852 (83.5%)	4,155 (59.5%)	487 (6,9%)	115 (1,6%)
						ASA + Ticagrelor 90 mg	7050	65.4 (8.4)	1,682 (23.9%)	1,187 (16.8%)	5,462 (77.5%)	2,241 (31.8%)	–	5,879 (83.0%)	4,190 (58.9%)	493 (7,0%)	127 (1,8%)
						ASA	7067	65.4 (8.3)	1,717 (24.3%)	1,143 (16.2%)	5,484 (77.6%)	2,257 (31.9%)	–	5,837 (82.6%)	4,213 (59.6%)	578 (8,2%)	54 (0,8%)
DAPT	August 2009	July 2011	Double-blinded, multicenter, placebo-controlled trial	Intention-to-treat	18	ASA + thienopyridine	5020	61.8 (10.2)	1,242 (24.7%)	1,222 (24.6%)	3,796 (75.8%)	1,556 (31.1%)	568 (11.3%)	1,518 (30.4%)	–	211 (4,2%)	119 (2,5%)
						ASA	4941	61.6 (10.1)	1,284 (26.0%)	1,210 (24.7%)	3,649 (74.0%)	1,481 (30.1%)	581 (11.8%)	1,529 (31.0%)	–	285 (5,8%)	73 (1,6%)

CABG, coronary artery bypass graft; PCI, percutaneous coronary intervention; MACE, major adverse cardiovascular events.

^a^ Age is reported as median (IQR) or mean (SD).

^b^ The modified intention-to-treat population included all patients who had undergone randomization after the exclusion of those who had been enrolled at a site that was closed before unblinding.

All domains analyzed on RoB 2 tool were judged as “low risk,” with exception of “Bias in the measurement of outcome” on the COMPASS trial bleeding outcome. This outcome was judged as “some concerns” due to lack of information regarding the simultaneous randomization to pantoprazole or placebo and the possible impact of that intervention on bleeding outcome analysis. Nevertheless, all RCTs showed low risk of bias (Supplementary Table S2).

### 3.1. Primary outcomes

**Figure 2:**
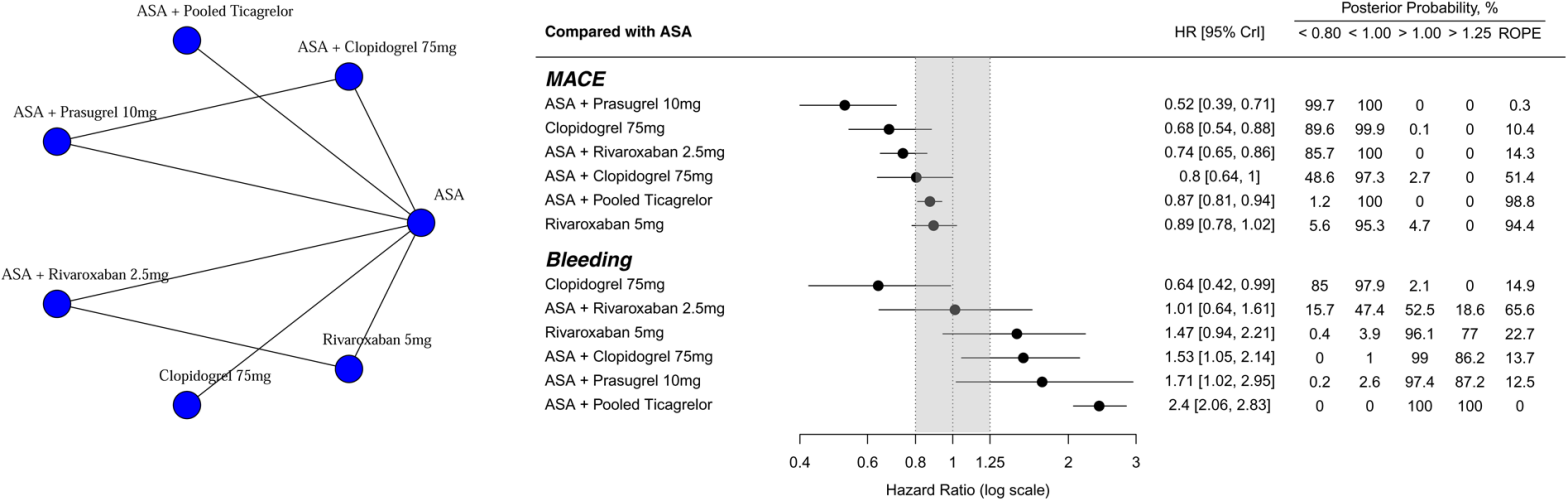
Network plot. Treatment effects compared with ASA on MACE and Bleeding outcomes, ordered according to underlying SUCRA values. HR below 1.0 favors the experimental treatment. On the left, treatment names are depicted. In the middle, forest plot shows each treatment effect median and 95% credible intervals. Gray area corresponds to the ROPE (from 0.8 to 1.25 HR). On the right, exact effect sizes along with posterior probabilities are shown. ROPE, region of practical equivalence; HR, hazard ratio.

The primary outcome occurred in 5,071 patients (6.29%) included in the analysis. Figure 2A depicts the network of the seven treatment regimens used in the MACE and bleeding outcome analysis. In this network, we pooled both ASA + ticagrelor dose regimens (60 and 90 mg), leading to an ASA + pooled ticagrelor node.

Compared with ASA, alternative antithrombotic treatments had a lower incidence of MACE and posterior probabilities of HR < 1.00 were greater than 95% (Figure 2B). Only three treatments (ASA + rivaroxaban 2.5 mg; clopidogrel 75 mg; ASA + prasugrel 10 mg) had posterior probabilities of HR < 0.80 greater than 85%. Rivaroxaban 5 mg and ASA + pooled ticagrelor presented with high probabilities (94.4% and 98.8%, respectively) of being practically equivalent to ASA, defined as an HR between 0.80 and 1.25 (ROPE, Figure 2B).

Regarding the bleeding outcome (Figure 2B), only Clopidogrel 75 mg was clearly superior to ASA [HR 0.65 (95% CrI: 0.42, 0.99); posterior probability of HR < 0.85 = 85%]. In contrast, ASA + pooled ticagrelor performed the worst [HR 2.40 (95% CrI: 2.06, 2.83); posterior probability of HR > 1.25 greater than 99.9%]. ASA + rivaroxaban 2.5 mg had the highest posterior probability of being equivalent to ASA (65.6%). League tables summarize all possible treatment comparisons for both MACE and bleeding outcomes [HRs (Supplementary Table S3); posterior probabilities (Supplementary Tables S4–S7)].

**Figure 3:**
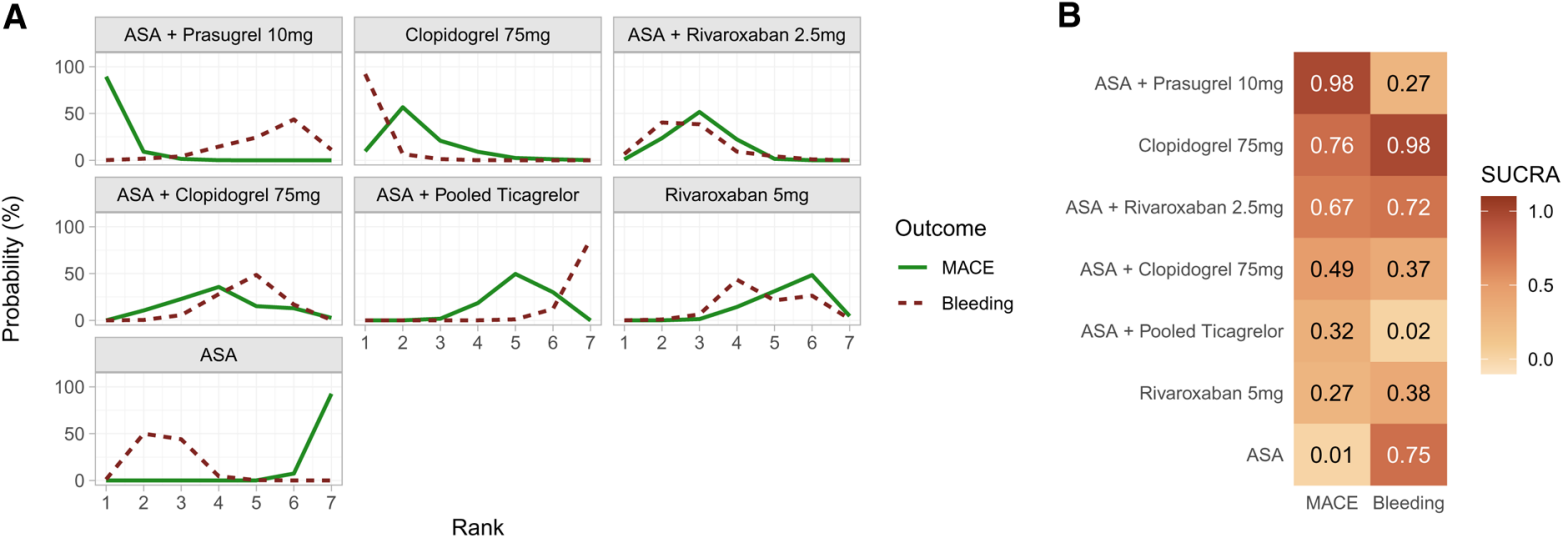
(**A**) ranking probabilities for MACE (solid line) and bleeding (dotted line) outcomes for each treatment. (**B**) heatmap with corresponding SUCRA values. While each row corresponds to a treatment, each column depicts one outcome.

Figure 3A shows the ranking probabilities and Figure 3B shows the SUCRA for each treatment option. Regarding MACE, ASA + prasugrel 10 mg performed the best (first rank probability = 89%; SUCRA = 0.98), and ASA performed the worst (last rank probability = 93%; SUCRA = 0.01). As expected, ranking probabilities and SUCRAs were different for the bleeding outcome, with clopidogrel 75 mg and ASA + pooled ticagrelor performing the best and worst, respectively. Complementing the estimates presented in Figure 3, the overall median ranks of each treatment along with the 95% CrI are depicted in Supplementary Figure S2.

Analyses separating ASA + pooled ticagrelor into different treatment nodes (ASA + ticagrelor 60 mg and ASA + ticagrelor 90 mg) are shown in Supplementary Figures S3–S6 and Tables S8–S12. When comparing HRs, posterior probabilities, ranking probabilities, SUCRAs, and median overall rank, ASA + ticagrelor 60 mg and ASA + ticagrelor 90 mg were similar [MACE: HR 0.85 (95% CrI: 0.76, 0.95) and posterior probability of HR < 1.00 = 99.8%; HR 0.85 (95% CrI: 0.76, 0.96); posterior probability of HR < 1.00 = 99.7%, respectively].

### 3.2. Secondary outcomes

Supplementary Figure S7 shows the networks for secondary outcomes [acute myocardial infarction (AMI), ischemic stroke, all-cause mortality, and cardiovascular-specific mortality]. The forest plot depicted in Supplementary Figure S8 summarizes these results comparing each experimental treatment to ASA, while Supplementary Tables S13–S27 depict the corresponding league tables. Regarding AMI, ASA + prasugrel 10 mg performed the best (SUCRA = 0.99). There were no available data on ASA + prasugrel 10 mg or ASA + clopidogrel 75 mg for the other three secondary outcomes. For these outcomes, ASA + rivaroxaban (2.5 mg) performed the best (SUCRA ranging from 0.90 to 0.99; Supplementary Figure S9). The overall median ranks of each treatment, along with the 95% CrI, are depicted in Supplementary Figure S10.

### 3.3. CINeMA

Confidence ratings of the treatment comparisons were judged as either low or very low. We found no concerns in most within-study bias and indirectness assessments (considering the data in Supplementary Figure S1 and Table S2). As shown in the league tables mentioned above Imprecision ranged from all categories of concerns (Supplementary Tables S3, S8, S13–S15). In turn, we considered the assessment of heterogeneity inapplicable because only fixed-effect meta-analyses were fitted; thus, there were no between-study variances or prediction intervals. Finally, we found major concerns regarding incoherence because all networks were “star-shaped,” lacking closed loops. Detailed CINeMA judgments for all treatment comparisons are shown in Supplementary Tables S28–S33.

## 4. Discussion

In this network meta-analysis, we found that the combination of prasugrel and ASA demonstrated the highest efficacy in preventing MACE (4). Clopidogrel monotherapy resulted in the lowest incidence of bleeding events (7). ASA + pooled ticagrelor was likely equivalent to ASA monotherapy in terms of efficacy, with the highest incidence of bleeding events (5, 8). In contrast to our study, the previous network meta-analysis of RCTs did not include the prasugrel + ASA arm, concluding that ASA + rivaroxaban 2.5 mg was the best evaluated treatment option. However, similar to our findings, ASA + ticagrelor resulted in reduced clinical benefit in their analysis (9).

Regarding secondary outcomes, ASA + prasugrel 10 mg demonstrated the highest efficacy in terms of AMI (4). Meanwhile, ASA + rivaroxaban 2.5 mg resulted in the lowest incidence of other secondary outcomes (ischemic stroke, all-cause mortality, and cardiovascular-specific mortality) (6). AMI findings might explain why our result differs from that of the previous meta-analysis (9), in which ASA + rivaroxaban 2.5 mg was found to be the best option in terms of MACE, motivated mostly by a reduced incidence of cerebrovascular events (6).

Prasugrel is recommended as the preferred therapy for acute coronary syndromes, and it is considered the best treatment option compared with ticagrelor and clopidogrel (26, 27). In pharmacodynamic studies of antiplatelet drugs, prasugrel resulted in reduced platelet aggregation and enhanced endothelial function. These mechanisms, in addition to the drug’s irreversible effect, might explain its efficacy in our study (28, 29). Despite ASA + prasugrel 10 mg being the most effective option in reducing MACE, the combination culminated in one of the least favorable safety profiles (4). This finding suggests that ASA prasugrel 10 mg may be suitable for patients with a high thrombotic risk but low bleeding risk.

Regarding safety outcomes, clopidogrel appeared to have better results, exhibiting a reduced bleeding risk, even when compared to ASA monotherapy (7). Moreover, it demonstrated protection against cardiovascular events, indicating a favorable balance between its safety profile and antithrombotic action (7). Through non-specific inhibition of cyclooxygenase, ASA increases the risk of gastrointestinal bleeding (30). This mechanism may explain why clopidogrel has a better safety profile than ASA in preventing MACE in patients with CCS.

Clopidogrel monotherapy also showed higher efficacy and safety compared to ASA + pooled ticagrelor (5, 7, 8). This finding differs from a previous meta-analysis published in 2021, possibly because it did not include the HOST-EXAM trial (31). Our results might have been affected by the pooled analysis of ticagrelor at different doses (90 mg or 60 mg twice daily), in addition to the fact that there was crossover between intervention arms; in this study several patients in the ticagrelor arm switch from 90 mg BI to 60 mg BID during the study (5).

Finally, the combination of ASA and low-dose rivaroxaban (2.5 mg BID) compared to ASA monotherapy demonstrated lower MACE^6^. Reductions in mortality and stroke as subcomponents may explain the overall reduction in MACE. ASA + rivaroxaban had higher bleeding rates than ASA monotherapy (7).

To our knowledge, this is the first network meta-analysis that included clopidogrel monotherapy and ASA + prasugrel. A previous study published in 2021 that aimed to establish the best long-term antithrombotic strategy in patients with CCS (31) differed from ours in the following points: 1- It did not include the most recent trial comparing AAS monotherapy to clopidogrel, the HOST-EXAM trial (7). This study, published in 2021, suggested that clopidogrel is an excellent alternative to ASA, especially in patients with a high bleeding risk; 2- it included the DAPT trial (4), but did not separate the analysis of ASA+ prasugrel from that of ASA + clopidogrel, and the first combination resulted in the greatest reduction in MACE in our study.

In addition, in contrast to recent meta- analyses (9, 31), we applied Bayesian methods. This statistical framework allowed us to estimate not only 95% CrIs, but also multiple actionable posterior probabilities. Herein, we estimated the probabilities of HR < 1.0, HR < 0.8, HR > 1.0, HR > 1.25, and of treatments being practically equivalent (HR between 0.8 and 1.25). These estimates allow readers to appreciate the results wholesomely and help to facilitate clinical decision making. Notably, ranking probabilities and SUCRAs have a more natural interpretation when estimated using Bayesian analyses.

We did not perform some analyses of interest due to a lack of data from the original studies, such as the analysis individualized by sex, the complexity of the lesions, or the type of stent used. The prevalence of women in these studies ranged from 20% to 31%, which may affect the bleeding risk analysis. Except for the DAPT study, in the other trials, the prevalence of multivessel coronary artery disease varies from 30% to 60%, which may affect the thrombotic risk analysis. Also, the follow-up period from single trials was relatively short for a chronic condition, varying from 18 to 39.9 months. Longer follow-up results should be reported in future studies.

## 5. Limitations

Our meta-analysis has some limitations. In the current analysis, although all selected RCTs included patients with CCS, some studies included more patients with diabetes and a history of CABG than others at different times from diagnosis. For example, one study included patients with multivessel arterial disease, a history of stable or unstable disease, previous percutaneous intervention, previous multi-vessel coronary artery bypass graft surgery, or even a history of myocardial infarction within 20 years (6).

Another potential limitation of our study is that our main analysis mixed results of different ticagrelor dosages (60 and 90 mg). We decided to pool these results because the THEMIS trial did not report data on patients treated with ASA + Ticagrelor 90 mg, only on pooled (ASA + Ticagrelor 60 mixed with 90 mg) or ASA + Ticagrelor 60 mg. To partially overcome this limitation, we performed sensitivity analyses in which we separately analyzed each Ticagrelor dosage. Results were very similar to pooled analyses and are available in the Supplementary material.

Notably, selected RCTs used in our analysis did not report outcomes according to ethnicity, which might have influenced the final results. For example, the better results obtained with the use of ASA + rivaroxaban 2.5 mg BID in Asian patients in a *post hoc* analysis is probably related to the hepatic metabolism of rivaroxaban and its increased anticoagulant action (32).

Finally, our safety endpoint was similar, but not homogenous in all trials included (Supplementary Table S1). This fact could have affected the bleeding outcome analysis.

## 6. Conclusion

In this study of RCTs evaluating antithrombotic therapy among patients with CCS, we found that combinations of ASA + prasugrel 10 mg and ASA + rivaroxaban 2.5 mg BID demonstrated the best efficacy results, mainly in reducing AMI, cerebrovascular events, and all-cause mortality. Clopidogrel monotherapy proved to be the option that best balances efficacy and safety. Compared with ASA, the combination of ASA and ticagrelor resulted in lower efficacy than clopidogrel monotherapy, although with the highest bleeding rates. However, low confidence ratings hamper definite conclusions. These results indicate that the current guidelines recommending ASA as monotherapy for patients with CCS need to be revised. RCTs incorporating strategies that individualize antithrombotic therapy according to patients' bleeding risk are needed to further close the outstanding gaps in CCS care.

## Data Availability

The original contributions presented in the study are included in the article/**Supplementary Material**, further inquiries can be directed to the corresponding author/s.
