# Supplementary material for: Antithrombotic regimens for the prevention of major adverse cardiac events in chronic coronary syndrome: A systematic review and network meta-analysis
Source: Front Cardiovasc Med. 2023 Apr 6;10:1040936. doi: 10.3389/fcvm.2023.1040936 (PMC10117905; doi:10.3389/fcvm.2023.1040936)

*Supplementary Material*

**Antithrombotic Regimens for the Prevention of Major Adverse Cardiac Events in Chronic Coronary Syndrome: A Systematic Review and Network Meta-Analysis**

**Table of Contents**

[Systematic Review](#_bookmark0) 4

[Search Strategies](#_bookmark1) . . . . . . . . . . . . . . . . . . . . . . . . . . . . . . . . . . . . 4

[Supplementary table 1: Trial definitions](#_bookmark2) . . . . . . . . . . . . . . . . . . . . . . . . . . . . . . . 5

[Supplementary figure 1: Effect Modifiers](#_bookmark3) . . . . . . . . . . . . . . . . . . . . . . . . . . . . . . . 6

[Supplementary table 2: Risk of Bias Assessment](#_bookmark4) . . . . . . . . . . . . . . . . . . . . . . . . . . . 7

[MACE](#_bookmark5) . . . . . . . . . . . . . . . . . . . . . . . . . . . . . . . . . . . . . . . 7

[Bleeding](#_bookmark6) . . . . . . . . . . . . . . . . . . . . . . . . . . . . . . . . . . . . . . 7

[Acute Myocardial Infarction](#_bookmark7) . . . . . . . . . . . . . . . . . . . . . . . . . . . 8

[Ischemic Stroke](#_bookmark8) . . . . . . . . . . . . . . . . . . . . . . . . . . . . . . . . . . 8

[All-cause Mortality](#_bookmark9) . . . . . . . . . . . . . . . . . . . . . . . . . . . . . . . . 8

[Cardiovascular Mortality](#_bookmark10) . . . . . . . . . . . . . . . . . . . . . . . . . . . . . 8

[Pooled Ticagrelor Networks: MACE and Bleeding Outcomes](#_bookmark13) 9

[League Tables](#_bookmark12) . . . . . . . . . . . . . . . . . . . . . . . . . . . . . . . . . . . . . . 9

[Supplementary table 3: Median and 95% Credible Intervals](#_bookmark11) . . . . . . . . . . . . . . . . . 9

[Supplementary table 4: Posterior Probabilities of Hazard Ratio < 1.0](#_bookmark14) 10

[Supplementary table 5: Posterior Probabilities of Hazard Ratio < 0.8](#_bookmark15) 11

[Supplementary table 6: Posterior Probabilities of Hazard Ratio > 1.25](#_bookmark16) 12

[Supplementary table 7: Posterior Probabilities of Hazard Ratio within the ROPE](#_bookmark17) 13

[Supplementary figure 2: Ranks with Uncertainty](#_bookmark18) 14

[Separate Dosages Ticagrelor Networks: MACE and Bleeding Outcomes](#_bookmark19) 15

[Supplementary figure 3: Network Plot](#_bookmark20) 15

[Supplementary figure 4: Forest Plot](#_bookmark21) 16

[League Tables](#_bookmark23) 17

[Supplementary table 8: Median and 95% Credible Intervals](#_bookmark22) 17

[Supplementary table 9: Posterior Probabilities of Hazard Ratio < 1.0](#_bookmark24) 18

[Supplementary table 10: Posterior Probabilities of Hazard Ratio < 0.8](#_bookmark25) 19

[Supplementary table 11: Posterior Probabilities of Hazard Ratio > 1.25](#_bookmark26) 20

[Supplementary table 12: Posterior Probabilities of Hazard Ratio within the ROPE](#_bookmark27) 21

[Supplementary figure 5: Ranks and SUCRA](#_bookmark28) 22

[Supplementary figure 6: Ranks with Uncertainty](#_bookmark29) 23

[Pooled Ticagrelor Networks: Secondary Outcomes](#_bookmark30) 24

[Supplementary figure 7: Network Plots](#_bookmark31) 24

[Supplementary figure 8: Forest Plot](#_bookmark32) 25

[League Tables](#_bookmark34) 26

[Supplementary table 13-15: Median and 95% Credible Intervals](#_bookmark33) 26

[Supplementary table 16-18: Posterior Probabilities of Hazard Ratio < 1.0](#_bookmark35) 28

[Supplementary table 19-21: Posterior Probabilities of Hazard Ratio < 0.8](#_bookmark36) 30

[Supplementary table 22-24: Posterior Probabilities of Hazard Ratio > 1.25](#_bookmark37) 32

[Supplementary table 25-27: Posterior Probabilities of Hazard Ratio within the ROPE](#_bookmark38) 34

[Supplementary figure 9: Ranks + SUCRA](#_bookmark39) 36

[Supplementary figure 10: Ranks with Uncertainty](#_bookmark40) 37

[CINeMA](#_bookmark43) 38

[Supplementary tables 28-33: Pooled Ticagrelor Networks](#_bookmark42) 38

[MACE](#_bookmark41) 38

[Bleeding](#_bookmark44) 39

[Acute Myocardial Infarction](#_bookmark45) 40

[Ischemic Stroke](#_bookmark47) 41

[All-cause Mortality](#_bookmark46) 41

[Cardiovascular Mortality](#_bookmark48) 42

[Supplementary tables 34-35: Separate Dosages Ticagrelor Networks](#_bookmark50) 43

[MACE](#_bookmark49) 43

[Bleeding](#_bookmark51) 44

# Systematic Review

## Search Strategies

Database Terms

**PubMed** (“Arteriosclerosis”[mh] OR “Angina, Stable”[mh]) AND (“Platelet Aggregation Inhibitors” [Mesh] OR “Dual Anti-Platelet Therapy” [Mesh] OR “Aspirin”[mh] OR “Clopidogrel”[mh] OR “Factor Xa Inhibitors”[mh] OR “apixaban”[Supplementary Concept] OR “edoxaban”[Supplementary Concept] OR “Rivaroxaban”[mh] OR “Purinergic P2Y Receptor Antagonists”[mh] OR “Ticagrelor”[mh] OR “Prasugrel”[tw] OR “Vorapaxar”[tw]) AND (“Myocardial Infarction”[mh] OR “Angina, Unstable”[mh] OR “Stroke”[Mesh] OR Death, Sudden, Cardiac [Mesh] OR “Myocardial Revascularization”[mh] OR “Major Adverse Cardiac Event”[tw] OR “Hemorrhage”[mh])

**EMBASE** (‘atherosclerosis’/exp OR ‘stable angina pectoris’/exp) AND (‘antithrombocytic agent’/exp

OR ‘dual antiplatelet therapy’/exp OR ‘acetylsalicylic acid’/mj OR ‘clopidogrel’/mj OR ‘blood clotting factor 10a inhibitor’/exp OR ‘apixaban’/mj OR ‘edoxaban’/mj OR ‘rivaroxaban’/mj OR ‘purinergic p2y receptor antagonist’/exp OR ‘ticagrelor’/mj OR ‘prasugrel’/mj OR ‘vorapaxar’/mj) AND (‘heart infarction’/mj OR ‘unstable angina pectoris’/mj OR ’cerebrovascular accident’/mj OR ‘heart muscle revascularization’/exp OR ‘major adverse cardiac event’/mj OR ‘bleeding’/exp)

CENTRAL /#1 MeSH descriptor: [Arteriosclerosis] explode all trees 11018 /#2 MeSH descriptor:

[Angina, Stable] explode all trees 360 /#3 #1 OR #2 11264 /#4 MeSH descriptor: [Platelet Aggregation Inhibitors] explode all trees 4016 /#5 MeSH descriptor: [Dual Anti-Platelet Therapy] explode all trees 45 /#6 MeSH descriptor: [Aspirin] explode all trees 6018 /#7 MeSH descriptor: [Clopidogrel] explode all trees 2074 /#8 MeSH descriptor: [Factor Xa Inhibitors] explode all trees 572 /#9 (apixaban):ti,ab,kw 984 /#10 (edoxaban):ti,ab,kw 598

/#11 MeSH descriptor: [Rivaroxaban] explode all trees 568 /#12 MeSH descriptor: [Purinergic P2Y Receptor Antagonists] explode all trees 359 /#13 MeSH descriptor: [Ticagrelor] explode all trees 786 /#14 #4 OR #5 OR #6 OR #7 OR #8 OR #9 OR #10 OR #11 OR #12 OR #13 10962 /#15 MeSH descriptor: [Myocardial Infarction] explode all trees 11333 /#16 MeSH descriptor: [Angina, Unstable] explode all trees 1138 /#17 MeSH descriptor: [Stroke] explode all trees 10342 /#18 MeSH descriptor: [Death, Sudden, Cardiac] explode all trees 635 /#19 MeSH descriptor: [Myocardial Revascularization] explode all trees 9254 /#20 (Major Adverse Cardiac Event):ti,ab,kw 2745 /#21 MeSH descriptor: [Hemorrhage] explode all trees 14754 /#22 #15 OR #16 OR #17 OR #18 OR #19 OR #20

OR #21 43672 **/#23 #3 AND #14 AND #22**

## Supplementary table 1: Trial definitions

Study MACE trial definition Safety endpoint definition

**DAPT** Composite of death, myocardial infarction,

or stroke during the randomized treatment period month 12 to month 30.

**COMPASS** Composite of cardiovascular death, stroke, or myocardial infarction.

**HOST-EXAM** Composite of cardiac death, non-fatal

myocardial infarction, ischaemic stroke, readmission due to acute coronary syndrome, and definite or probable stent thrombosis (originally referred to as "thrombotic composite outcome").

**THEMIS** Composite of cardiovascular death,

myocardial infarction, or stroke.

**PEGASUS** Composite of cardiovascular death,

myocardial infarction, or stroke.

Incidence of moderate or severe bleeding during the randomized treatment period month 12 to month 30 assessed according to the Global Utilization of Streptokinase and Tissue Plasminogen Activator for Occluded Arteries [GUSTO] criteria.* Modification of the International Society on Thrombosis and Haemostasis (ISTH) criteria for major bleeding and included fatal bleeding, symptomatic bleeding into a critical organ, bleeding into a surgical site requiring reoperation, and bleeding that led to hospitalization (including presentation to an acute care facility without an overnight stay). Unlike the ISTH criteria, we considered all bleeding that led to presentation to an acute care facility or

hospitalization as major.

Major bleeding, that was defined as Bleeding Academic Research Consortium (BARC) type bleeding of least 3.**

TIMI major bleeding. ***

Major bleeding, which was defined according to the TIMI classification.***

* Moderate bleeding is defined as bleeding requiring blood transfusion, but not resulting in hemodynamic compromise, and severe bleeding is defined as either an intracerebral hemorrhage or bleeding resulting in substantial hemodynamic compromise requiring treatment according to the GUSTO criteria.

** BARC type bleeding of 3 is defined as clinical, laboratory, and/or imaging evidence of bleeding with healthcare responses, as listed: Any transfusion with overt bleeding; Overt bleeding plus hemoglobin (Hb) drop >=3 to <5 g/dI (provided Hb drop is related to bleeding); Overt bleeding plus Hb drop >=5 g/dl; Cardiac tamponade; Bleeding requiring surgical intervention for control; Bleeding requiring intravenous vasoactive drugs; Intracranial hemorrhage; Intraocular bleed compromising vision.

*** TIMI major bleeding is defined as any intracranial bleeding (excluding microhemorrhages <10 mm evi- dent only on gradient-echo MRI), or clinically overt signs of hemorrhage associated with a drop in hemoglobin of >=5 g/dL or a >=15% absolute decrease in haematocrit, or a fatal bleeding (bleeding that directly results in death within 7 d).

##


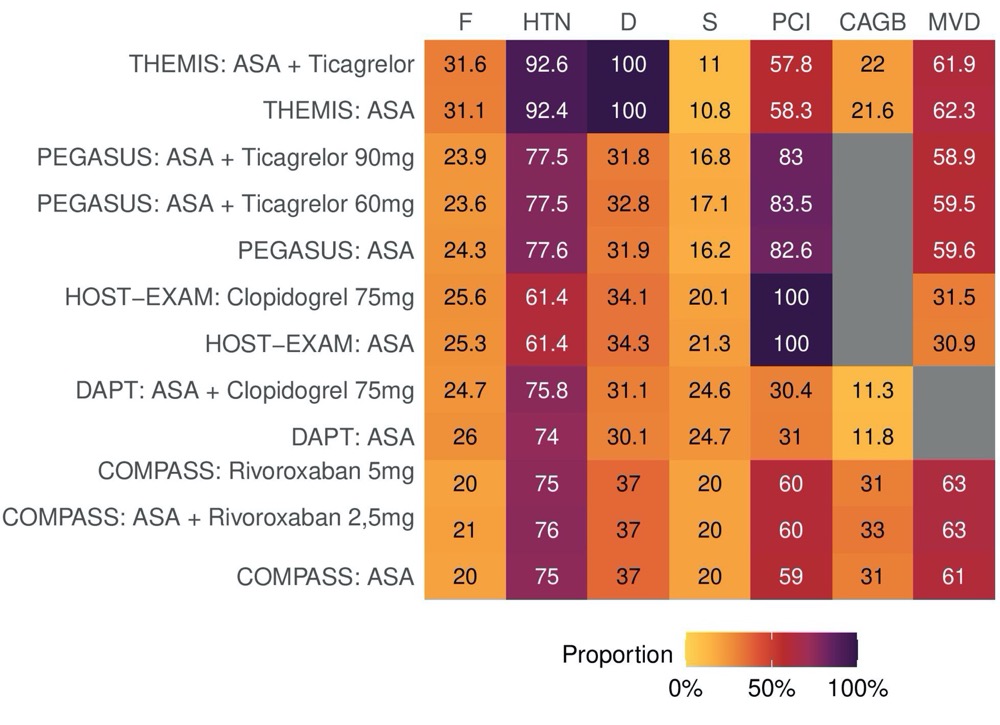


## Supplementary figure 1: Effect Modifiers

Heatmap on the proportions of potential efffect modifiers across included studies and treat- ment arms. Each row corresponds to a specific study and treatment arm combination. Each column corresponds to a specific effect modifier. The exact proportion is depicted by both filled color and the number on top in each cell.

Abbreviations: F, female; HTN, hypertension; D, diabetes; S, smoker; PCI, percutaneous coronary intervention; CAGB, coronary artery bypass graft; MVD, multivessel disease.

## Supplementary Table 2: Risk of Bias Assessment

Risk of bias for domain-level as well as overall judgements.s

D1: bias arising from randomisation process; D2: bias arising from deviations from intended interventions; D3: bias due to missing outcome data; D4: bias in measurement of the outcome; D5: bias in selection of the reported result.

### MACE

###
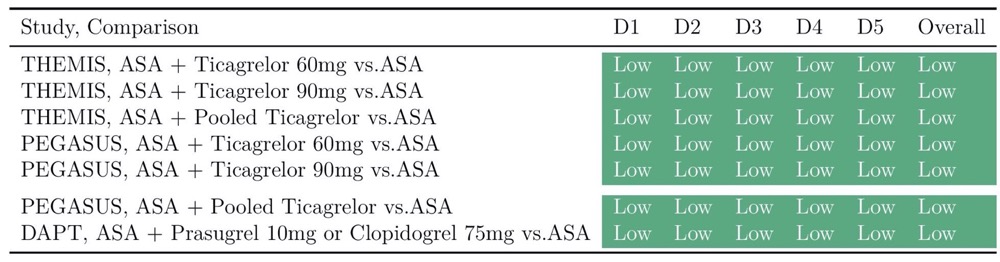


### Bleeding

###
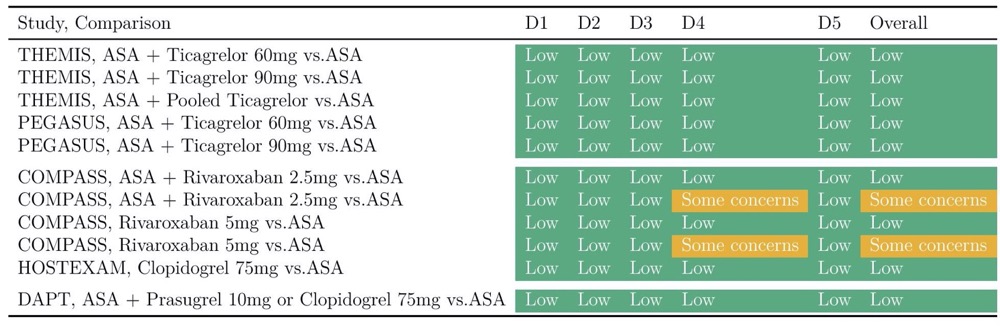


### Acute Myocardial Infarction


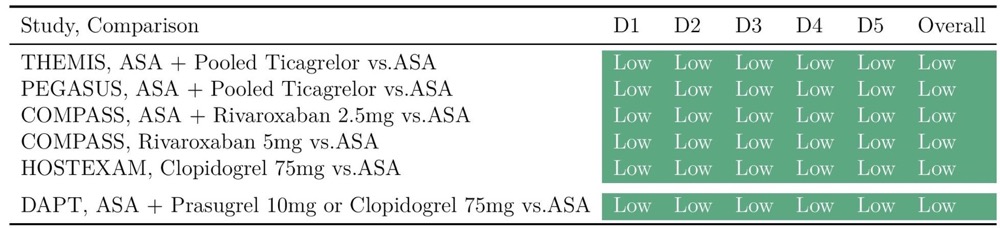


### Ischemic Stroke


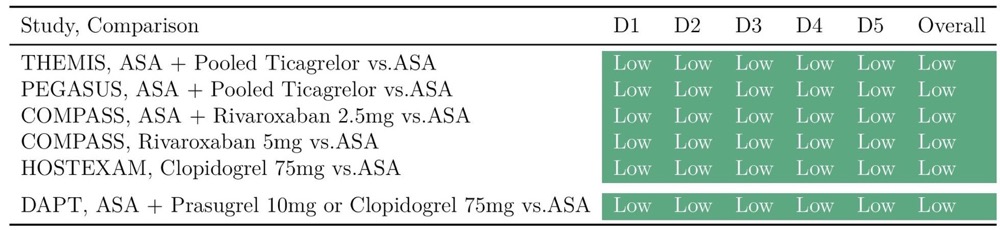


### All-cause Mortality

###
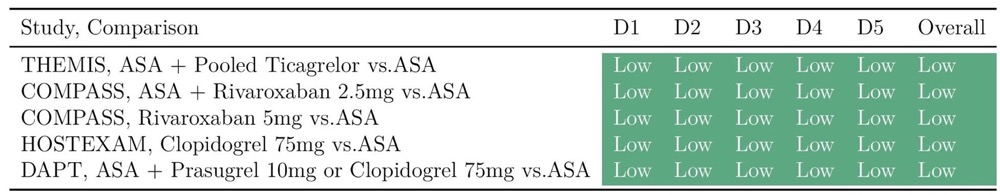


### Cardiovascular Mortality


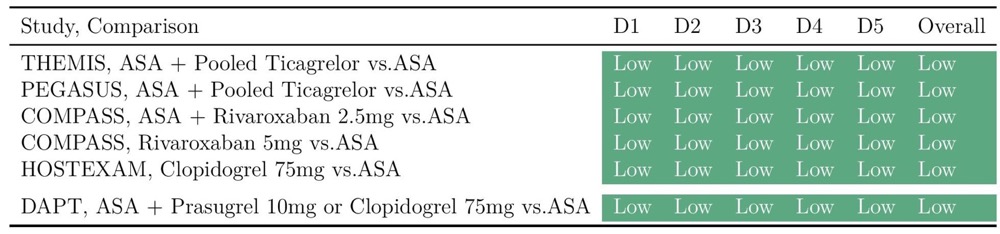


# Pooled Ticagrelor Networks: MACE and Bleeding Outcomes

9

## League Tables

### Supplementary Table 3: Median and 95% Credible Intervals

| ASA | 0.89 (0.78, 1.02) | 0.68 (0.54, 0.88) | 0.74 (0.65, 0.86) | 0.52 (0.39, 0.71) | 0.80 (0.64, 1.00) | 0.87 (0.81, 0.94) |
| --- | --- | --- | --- | --- | --- | --- |
| 1.47 (0.94, 2.21) | Rivaroxaban 5mg | 0.76 (0.57, 1.01) | 0.83 (0.73, 0.96) | 0.59 (0.42, 0.81) | 0.90 (0.70, 1.18) | 0.98 (0.84, 1.14) |
| 0.64 (0.42, 0.99) | 0.44 (0.24, 0.77) | Clopidogrel 75mg | 1.09 (0.81, 1.44) | 0.77 (0.52, 1.15) | 1.18 (0.85, 1.66) | 1.28 (0.97, 1.64) |
| 1.01 (0.64, 1.61) | 0.69 (0.44, 1.09) | 1.58 (0.85, 2.97) | ASA + Rivaroxaban 2.5mg | 0.71 (0.50, 0.99) | 1.08 (0.83, 1.42) | 1.18 (1.01, 1.38) |
| 1.71 (1.02, 2.95) | 1.16 (0.60, 2.34) | 2.67 (1.33, 5.18) | 1.68 (0.84, 3.39) | ASA + Prasugrel 10mg | 1.53 (1.18, 1.97) | 1.67 (1.19, 2.24) |
| 1.53 (1.05, 2.14) | 1.04 (0.58, 1.77) | 2.39 (1.37, 4.22) | 1.50 (0.82, 2.64) | 0.89 (0.60, 1.40) | ASA + Clopidogrel 75mg | 1.09 (0.87, 1.40) |
| 2.40 (2.06, 2.83) | 1.63 (1.04, 2.59) | 3.76 (2.42, 6.14) | 2.37 (1.45, 3.85) | 1.41 (0.80, 2.43) | 1.58 (1.07, 2.36) | Pooled Ticagrelor |

Hazard ratios (95% credible interval) for the MACE or Bleeding outcomes. Treatments are shown in the diagonal. Results to the right of this diagonal (right upper half table) correspond to the MACE outcome. Results to the left (left lower half table) correspond to the Bleeding outcome. Comparisons between treatments should be read from left to right and the estimate is in the cell in common between the column-defining treatment and the row-defining treatment. For both outcomes, a hazard ratio < 1.0 favors the column-defining treatment. For example, in the Rivaroxaban 5mg vs. ASA comparison for MACE, the corresponding HR (95% CrI) was 0.89 (0.78, 1.02), favoring Rivaroxaban 5mg (ie, HR < 1.0).

10

### Supplementary table 4: Posterior Probabilities of Hazard Ratio < 1.0

| ASA | 95.31 | 99.88 | 100.00 | 100.00 | 97.26 | 100.00 |
| --- | --- | --- | --- | --- | --- | --- |
| 3.92 | Rivaroxaban 5mg | 96.90 | 99.54 | 99.91 | 78.17 | 61.26 |
| 97.92 | 99.76 | Clopidogrel 75mg | 28.56 | 89.94 | 16.98 | 3.16 |
| 47.45 | 94.46 | 7.05 | ASA + Rivaroxaban 2.5mg | 97.74 | 27.98 | 2.17 |
| 2.57 | 33.59 | 0.25 | 7.61 | ASA + Prasugrel 10mg | 0.00 | 0.06 |
| 1.03 | 44.61 | 0.05 | 8.58 | 69.67 | ASA + Clopidogrel 75mg | 25.49 |
| 0.00 | 1.73 | 0.00 | 0.05 | 11.50 | 1.25 | Pooled Ticagrelor |

Posterior probabilities (%) of hazard ratio < 1.0 for the MACE or Bleeding outcomes. Treatments are shown in the diagonal. Results to the right of this diagonal (right upper half table) correspond to the MACE outcome. Results to the left (left lower half table) correspond to the Bleeding outcome. Comparisons between treatments should be read from left to right and the estimate is in the cell in common between the column-defining treatment and the row-defining treatment. For both outcomes, a hazard ratio < 1.0 favors the column-defining treatment. For example, in the Rivaroxaban 5mg vs. ASA comparison for MACE, there was a posterior probability of 95.31% that the hazard ratio is below 1.0 (ie, there was a 95.31% probability for Rivaroxaban 5mg superiority).

11

### Supplementary table 5: Posterior Probabilities of Hazard Ratio < 0.8

| ASA | 5.58 | 89.61 | 85.65 | 99.66 | 48.62 | 1.16 |
| --- | --- | --- | --- | --- | --- | --- |
| 0.35 | Rivaroxaban 5mg | 62.45 | 29.80 | 96.12 | 18.44 | 0.56 |
| 85.04 | 97.92 | Clopidogrel 75mg | 1.82 | 57.90 | 1.14 | 0.01 |
| 15.74 | 73.61 | 1.47 | ASA + Rivaroxaban 2.5mg | 75.70 | 1.14 | 0.00 |
| 0.24 | 14.39 | 0.04 | 2.01 | ASA + Prasugrel 10mg | 0.00 | 0.00 |
| 0.05 | 18.09 | 0.00 | 1.71 | 29.99 | ASA + Clopidogrel 75mg | 0.64 |
| 0.00 | 0.06 | 0.00 | 0.00 | 2.23 | 0.05 | Pooled Ticagrelor |

Posterior probabilities (%) of hazard ratio < 0.8 for the MACE or Bleeding outcomes. Treatments are shown in the diagonal. Results to the right of this diagonal (right upper half table) correspond to the MACE outcome. Results to the left (left lower half table) correspond to the Bleeding outcome. Comparisons between treatments should be read from left to right and the estimate is in the cell in common between the column-defining treatment and the row-defining treatment. For both outcomes, a hazard ratio < 1.0 favors the column-defining treatment. For example, in the Rivaroxaban 5mg vs. ASA comparison for MACE, there was a posterior probability of 5.58% that the hazard ratio is below 0.8 (ie, there was a 5.58% probability for Rivaroxaban 5mg superiority).

12

### Supplementary table 6: Posterior Probabilities of Hazard Ratio > 1.25

| ASA | 0.00 | 0.00 | 0.00 | 0.00 | 0.01 | 0.00 |
| --- | --- | --- | --- | --- | --- | --- |
| 76.95 | Rivaroxaban 5mg | 0.03 | 0.00 | 0.00 | 0.57 | 0.07 |
| 0.05 | 0.00 | Clopidogrel 75mg | 17.11 | 0.94 | 36.49 | 57.04 |
| 18.64 | 0.59 | 76.92 | ASA + Rivaroxaban 2.5mg | 0.05 | 14.91 | 22.07 |
| 87.24 | 41.85 | 98.52 | 79.15 | ASA + Prasugrel 10mg | 93.88 | 95.88 |
| 86.21 | 25.57 | 98.76 | 72.86 | 6.39 | ASA + Clopidogrel 75mg | 12.20 |
| 100.00 | 87.86 | 100.00 | 99.36 | 66.38 | 87.86 | Pooled Ticagrelor |

Posterior probabilities (%) of hazard ratio > 1.25 for the MACE or Bleeding outcomes. Treatments are shown in the diagonal. Results to the right of this diagonal (right upper half table) correspond to the MACE outcome. Results to the left (left lower half table) correspond to the Bleeding outcome. Comparisons between treatments should be read from left to right and the estimate is in the cell in common between the column-defining treatment and the row-defining treatment. For both outcomes, a hazard ratio < 1.0 favors the column-defining treatment. For example, in the Rivaroxaban 5mg vs. ASA comparison for MACE, there was a posterior probability of 0.00% that the hazard ratio is above 1.25.

13

### Supplementary table 7: Posterior Probabilities of Hazard Ratio within the ROPE

| ASA | 94.42 | 10.39 | 14.35 | 0.34 | 51.36 | 98.84 |
| --- | --- | --- | --- | --- | --- | --- |
| 22.70 | Rivaroxaban 5mg | 37.52 | 70.20 | 3.88 | 80.99 | 99.36 |
| 14.91 | 2.08 | Clopidogrel 75mg | 81.06 | 41.16 | 62.38 | 42.95 |
| 65.62 | 25.80 | 21.60 | ASA + Rivaroxaban 2.5mg | 24.25 | 83.95 | 77.92 |
| 12.53 | 43.76 | 1.44 | 18.84 | ASA + Prasugrel 10mg | 6.12 | 4.12 |
| 13.74 | 56.34 | 1.24 | 25.42 | 63.62 | ASA + Clopidogrel 75mg | 87.16 |
| 0.00 | 12.07 | 0.00 | 0.64 | 31.40 | 12.09 | Pooled Ticagrelor |

Posterior probabilities (%) of hazard ratio within the region of practical equivalence (ROPE), ie, between 0.80 and 1.25, for the MACE or Bleeding outcomes. Treatments are shown in the diagonal. Results to the right of this diagonal (right upper half table) correspond to the MACE outcome. Results to the left (left lower half table) correspond to the Bleeding outcome. Comparisons between treatments should be read from left to right and the estimate is in the cell in common between the column-defining treatment and the row-defining treatment. For both outcomes, a hazard ratio < 1.0 favors the column-defining treatment. For example, in the Rivaroxaban 5mg vs. ASA comparison for MACE, there was a posterior probability of 94.42% that the hazard ratio is within the ROPE.

## Supplementary figure 2: Ranks with Uncertainty

ASA + Prasugrel 10mg


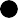

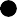

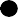

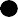

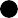

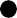

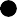


MACE


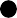

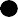

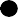

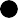

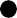

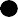

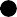


Bleeding

Clopidogrel 75mg

ASA + Rivaroxaban 2.5mg

ASA + Clopidogrel 75mg

Pooled Ticagrelor

Rivaroxaban 5mg

ASA

1 2 3 4 5 6 7 1 2 3 4 5 6 7

Rank

Marginal posterior distributions for the rank of each treatment. Left panel depicts ranks for MACE, while right panel for the Bleeding outcome. In each panel, there are a point esti- mate, interval bar, and bar plot for each treatment (Y-axis). The point estimate represents the median rank. The interval bar shows the 95% credible (quantile) interval of the under- lying marginal posterior distribution. Lastly, the bar plot shows the cumulative distribution function (CDF).

# Separate Dosages Ticagrelor Networks: MACE and Bleeding Outcomes

## eFigure 3: Network Plot

ASA + Prasugrel 10mg

ASA + Rivaroxaban 2.5mg


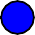

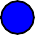

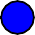

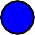

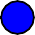

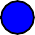

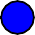

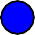


ASA + Ticagrelor 60mg

ASA + Ticagrelor 90mg

ASA + Clopidogrel 75mg

ASA

Rivaroxaban 5mg

Clopidogrel 75mg

## Supplementary figure 4: Forest Plot


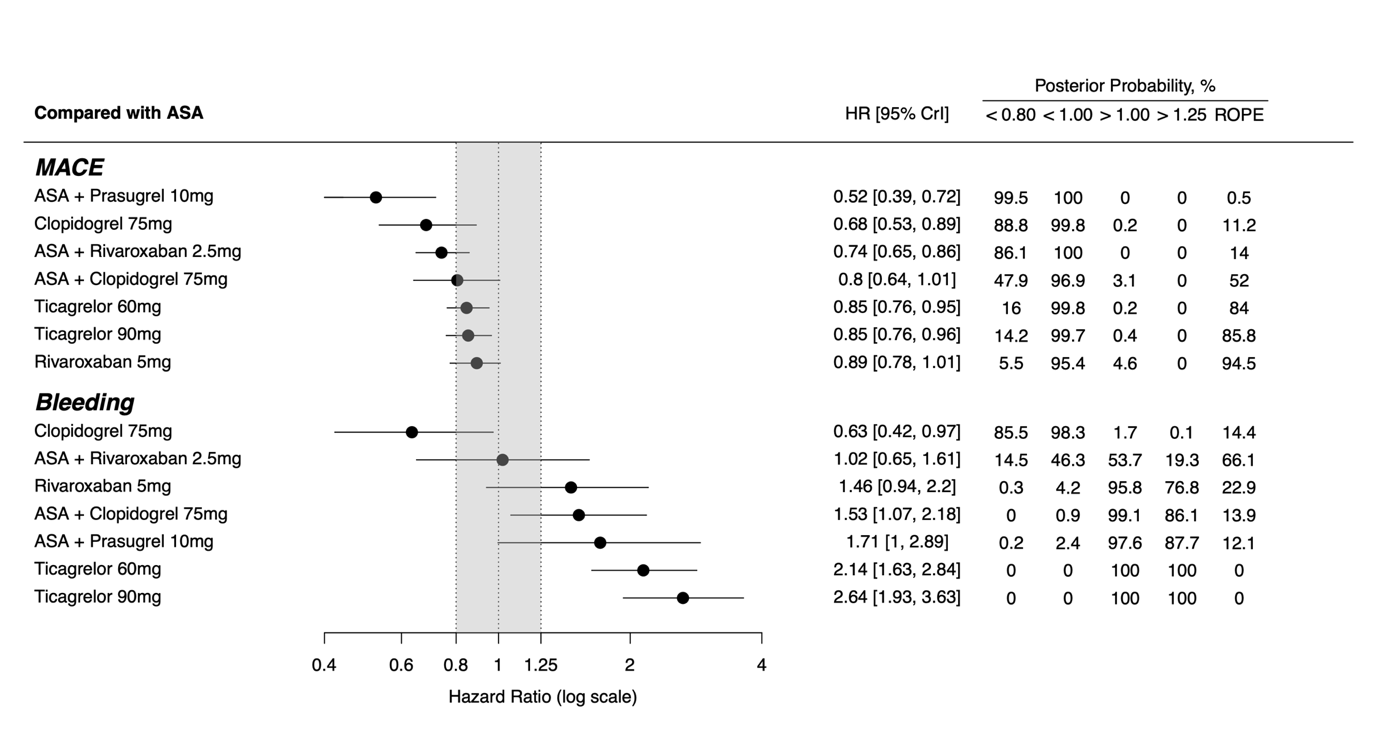


16

Left panel: Network of MACE and Bleeding outcomes while separing Ticagrelor’s dosages. Right panel: Treatment effects compared to ASA on MACE and Bleeding outcomes, ordered according to underlying SUCRA values. HR below 1.0 favors the experimental treatment. On the left, treatment names are depicted. In the middle, forest plot shows each treatment effect median and 95% credible intervals. Gray area corresponds to the ROPE (from 0.8 to 1.25 HR). On the right, exact effect sizes along with posterior probabilities are shown. Abbreviations: ROPE, region of practical equivalence; HR, hazard ratio.

17

## League Tables

### eTable 8: Median and 95% Credible Intervals

| ASA | 0.89 (0.78, 1.01) | 0.85 (0.76, 0.95) | 0.68 (0.53, 0.89) | 0.85 (0.76, 0.96) | 0.74 (0.65, 0.86) | 0.52 (0.39, 0.72) | 0.80 (0.64, 1.01) |
| --- | --- | --- | --- | --- | --- | --- | --- |
| 1.46 (0.94, 2.20) | Rivaroxaban 5mg | 0.95 (0.80, 1.14) | 0.77 (0.58, 1.02) | 0.96 (0.79, 1.14) | 0.83 (0.72, 0.95) | 0.59 (0.42, 0.83) | 0.90 (0.69, 1.17) |
| 2.14 (1.63, 2.84) | 1.46 (0.88, 2.46) | Ticagrelor 60mg | 0.81 (0.61, 1.07) | 1.01 (0.90, 1.14) | 0.88 (0.73, 1.04) | 0.62 (0.45, 0.87) | 0.95 (0.74, 1.22) |
| 0.63 (0.42, 0.97) | 0.43 (0.24, 0.80) | 0.30 (0.18, 0.49) | Clopidogrel 75mg | 1.25 (0.94, 1.66) | 1.09 (0.81, 1.45) | 0.77 (0.52, 1.16) | 1.18 (0.83, 1.65) |
| 2.64 (1.93, 3.63) | 1.80 (1.07, 3.14) | 1.23 (0.83, 1.86) | 4.17 (2.46, 6.83) | Ticagrelor 90mg | 0.87 (0.72, 1.04) | 0.61 (0.45, 0.88) | 0.94 (0.73, 1.23) |
| 1.02 (0.65, 1.61) | 0.70 (0.38, 1.28) | 0.48 (0.28, 0.81) | 1.61 (0.87, 2.97) | 0.39 (0.23, 0.68) | ASA + Rivaroxaban 2.5mg | 0.71 (0.51, 1.01) | 1.08 (0.84, 1.42) |
| 1.71 (1.00, 2.89) | 1.17 (0.60, 2.28) | 0.80 (0.44, 1.43) | 2.68 (1.40, 5.31) | 0.64 (0.35, 1.22) | 1.68 (0.86, 3.35) | ASA + Prasugrel 10mg | 1.54 (1.18, 1.97) |
| 1.53 (1.07, 2.18) | 1.04 (0.57, 1.74) | 0.71 (0.45, 1.10) | 2.41 (1.33, 4.10) | 0.58 (0.37, 0.93) | 1.50 (0.84, 2.66) | 0.90 (0.51, 1.61) | ASA + Clopidogrel 75mg |

Hazard ratios (95% credible interval) for the MACE or Bleeding outcomes. Treatments are shown in the diagonal. Results to the right of this diagonal (right upper half table) correspond to the MACE outcome. Results to the left (left lower half table) correspond to the Bleeding outcome. Comparisons between treatments should be read from left to right and the estimate is in the cell in common between the column-defining treatment and the row-defining treatment. For both outcomes, a hazard ratio < 1.0 favors the column-defining treatment. For example, in the Rivaroxaban 5mg vs. ASA comparison for MACE, the corresponding HR (95% CrI) was 0.89 (0.78, 1.01), favoring Rivaroxaban 5mg (ie, HR < 1.0).

18

### Supplementary table 9: Posterior Probabilities of Hazard Ratio < 1.0

| ASA | 95.41 | 99.83 | 99.80 | 99.65 | 100.00 | 100.00 | 96.94 |
| --- | --- | --- | --- | --- | --- | --- | --- |
| 4.19 | Rivaroxaban 5mg | 72.45 | 96.55 | 68.62 | 99.60 | 99.81 | 77.71 |
| 0.00 | 7.22 | Ticagrelor 60mg | 93.51 | 44.66 | 92.74 | 99.75 | 65.62 |
| 98.30 | 99.67 | 100.00 | Clopidogrel 75mg | 6.05 | 29.04 | 89.31 | 17.41 |
| 0.00 | 1.74 | 15.07 | 0.00 | Ticagrelor 90mg | 93.29 | 99.72 | 67.73 |
| 46.27 | 87.56 | 99.69 | 6.48 | 99.95 | ASA + Rivaroxaban 2.5mg | 97.45 | 27.45 |
| 2.43 | 32.60 | 76.79 | 0.10 | 91.61 | 7.49 | ASA + Prasugrel 10mg | 0.04 |
| 0.86 | 44.02 | 92.47 | 0.09 | 98.98 | 8.45 | 64.12 | ASA + Clopidogrel 75mg |

Hazard ratios (95% credible interval) for the MACE or Bleeding outcomes. Treatments are shown in the diagonal. Results to the right of this diagonal (right upper half table) correspond to the MACE outcome. Results to the left (left lower half table) correspond to the Bleeding outcome. Comparisons between treatments should be read from left to right and the estimate is in the cell in common between the column-defining treatment and the row-defining treatment. For both outcomes, a hazard ratio < 1.0 favors the column-defining treatment. For example, in the Rivaroxaban 5mg vs. ASA comparison for MACE, the corresponding HR (95% CrI) was 95.41, favoring Rivaroxaban 5mg (ie, HR < 1.0).

19

### Supplementary table 10: Posterior Probabilities of Hazard Ratio < 0.8

| ASA | 5.53 | 16.05 | 88.83 | 14.24 | 86.05 | 99.50 | 47.95 |
| --- | --- | --- | --- | --- | --- | --- | --- |
| 0.27 | Rivaroxaban 5mg | 2.69 | 61.99 | 2.41 | 28.99 | 96.06 | 18.36 |
| 0.00 | 1.16 | Ticagrelor 60mg | 47.15 | 0.00 | 15.75 | 93.44 | 8.50 |
| 85.49 | 97.67 | 100.00 | Clopidogrel 75mg | 0.11 | 1.98 | 56.94 | 1.32 |
| 0.00 | 0.14 | 1.93 | 0.00 | Ticagrelor 90mg | 19.01 | 94.04 | 9.84 |
| 14.54 | 67.14 | 97.06 | 1.30 | 99.52 | ASA + Rivaroxaban 2.5mg | 75.20 | 1.12 |
| 0.22 | 13.63 | 50.51 | 0.04 | 74.86 | 1.79 | ASA + Prasugrel 10mg | 0.00 |
| 0.00 | 17.41 | 68.16 | 0.01 | 90.64 | 1.70 | 35.40 | ASA + Clopidogrel 75mg |

Posterior probabilities (%) of hazard ratio < 0.8 for the MACE or Bleeding outcomes. Treatments are shown in the diagonal. Results to the right of this diagonal (right upper half table) correspond to the MACE outcome. Results to the left (left lower half table) correspond to the Bleeding outcome. Comparisons between treatments should be read from left to right and the estimate is in the cell in common between the column-defining treatment and the row-defining treatment. For both outcomes, a hazard ratio < 1.0 favors the column-defining treatment. For example, in the Rivaroxaban 5mg vs. ASA comparison for MACE, there was a posterior probability of 5.53% that the hazard ratio is below 0.8 (ie, there was a 5.53% probability for Rivaroxaban 5mg superiority).

20

### Supplementary table 11: Posterior Probabilities of Hazard Ratio > 1.25

| ASA | 0.00 | 0.00 | 0.00 | 0.00 | 0.00 | 0.00 | 0.03 |
| --- | --- | --- | --- | --- | --- | --- | --- |
| 76.79 | Rivaroxaban 5mg | 0.10 | 0.04 | 0.19 | 0.00 | 0.00 | 0.84 |
| 100.00 | 72.41 | Ticagrelor 60mg | 0.11 | 0.00 | 0.00 | 0.00 | 1.68 |
| 0.06 | 0.01 | 0.00 | Clopidogrel 75mg | 49.40 | 17.16 | 0.92 | 36.89 |
| 100.00 | 91.36 | 47.58 | 100.00 | Ticagrelor 90mg | 0.03 | 0.00 | 1.74 |
| 19.31 | 3.04 | 0.03 | 79.15 | 0.00 | ASA + Rivaroxaban 2.5mg | 0.11 | 15.01 |
| 87.69 | 41.61 | 6.88 | 98.60 | 2.02 | 79.42 | ASA + Prasugrel 10mg | 94.09 |
| 86.11 | 26.52 | 0.73 | 98.95 | 0.06 | 72.36 | 12.75 | ASA + Clopidogrel 75mg |

Posterior probabilities (%) of hazard ratio < 0.8 for the MACE or Bleeding outcomes. Treatments are shown in the diagonal. Results to the right of this diagonal (right upper half table) correspond to the MACE outcome. Results to the left (left lower half table) correspond to the Bleeding outcome. Comparisons between treatments should be read from left to right and the estimate is in the cell in common between the column-defining treatment and the row-defining treatment. For both outcomes, a hazard ratio < 1.0 favors the column-defining treatment. For example, in the Rivaroxaban 5mg vs. ASA comparison for MACE, there was a posterior probability of 0.00% that the hazard ratio is below 0.8 (ie, there was a 0.00% probability for Rivaroxaban 5mg superiority).

21

### Supplementary table 12: Posterior Probabilities of Hazard Ratio within the ROPE

| ASA | 94.47 | 83.95 | 11.18 | 85.76 | 13.95 | 0.50 | 52.02 |
| --- | --- | --- | --- | --- | --- | --- | --- |
| 22.94 | Rivaroxaban 5mg | 97.21 | 37.97 | 97.40 | 71.01 | 3.94 | 80.80 |
| 0.00 | 26.42 | Ticagrelor 60mg | 52.74 | 100.00 | 84.25 | 6.56 | 89.83 |
| 14.45 | 2.31 | 0.00 | Clopidogrel 75mg | 50.49 | 80.86 | 42.14 | 61.79 |
| 0.00 | 8.50 | 50.50 | 0.00 | Ticagrelor 90mg | 80.96 | 5.96 | 88.42 |
| 66.15 | 29.83 | 2.91 | 19.55 | 0.47 | ASA + Rivaroxaban 2.5mg | 24.69 | 83.86 |
| 12.09 | 44.76 | 42.61 | 1.36 | 23.11 | 18.79 | ASA + Prasugrel 10mg | 5.91 |
| 13.89 | 56.06 | 31.11 | 1.04 | 9.30 | 25.94 | 51.85 | ASA + Clopidogrel 75mg |

Posterior probabilities (%) of hazard ratio within the region of practical equivalence (ROPE), ie, between 0.80 and 1.25, for the MACE or Bleeding outcomes. Treatments are shown in the diagonal. Results to the right of this diagonal (right upper half table) correspond to the MACE outcome. Results to the left (left lower half table) correspond to the Bleeding outcome. Comparisons between treatments should be read from left to right and the estimate is in the cell in common between the column-defining treatment and the row-defining treatment. For both outcomes, a hazard ratio < 1.0 favors the column-defining treatment. For example, in the Rivaroxaban 5mg vs. ASA comparison for MACE, there was a posterior probability of 94.47% that the hazard ratio is within the ROPE.

## Supplementary Figure 5: Ranks and SUCRA


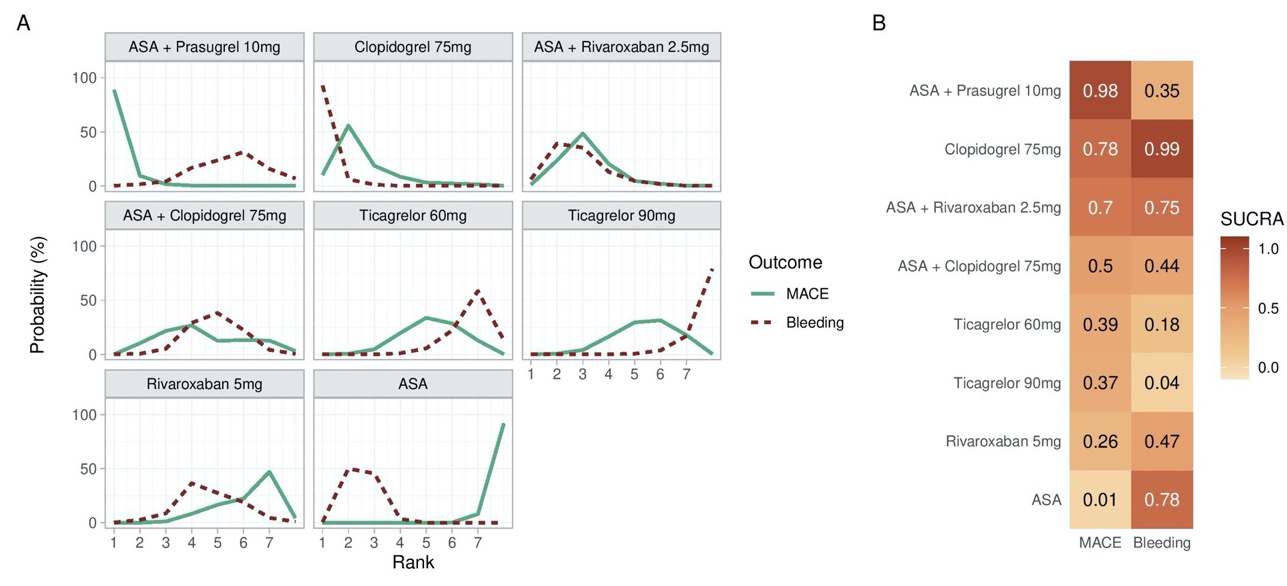


22

Panel A: Ranking probabilities for MACE (solid line) and Bleeding (dotted line) outcomes for each treatment. Panel B: Heatmap with corresponding SUCRA values. While each row corresponds to a treatment, each column depicts one outcome.

## Supplementary figure 6: Ranks with Uncertainty

ASA + Prasugrel 10mg


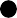

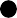

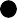

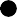

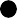

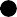

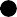

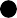


MACE


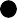

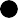

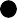

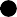

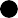

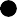

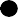

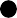


Bleeding

Clopidogrel 75mg

ASA + Rivaroxaban 2.5mg

ASA + Clopidogrel 75mg

Ticagrelor 60mg

Ticagrelor 90mg

Rivaroxaban 5mg

ASA

1 2 3 4 5 6 7 8 1 2 3 4 5 6 7 8

Rank

Marginal posterior distributions for the rank of each treatment. Left panel depicts ranks for MACE, while right panel for the Bleeding outcome. In each panel, there are a point estimate, interval bar, and bar plot for each treatment (Y-axis). The point estimate represents the median rank. The interval bar shows the 95% credible interval of the underlying marginal posterior distribution. Lastly, the bar plot shows the cumulative distribution function (CDF).

# Pooled Ticagrelor Networks: Secondary Outcomes

## Supplementary figure 7: Network Plots

**Acute Myocardial Infarction**

ASA + Pooled Ticagrelor


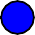

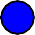

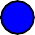

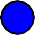

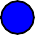

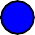

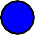


ASA + Clopidogrel 75mg

ASA + Prasugrel 10mg

ASA

ASA + Rivaroxaban 2.5mg

Rivaroxaban 5mg

Clopidogrel 75mg

**Ischemic Stroke, All-cause Mortality, and Cardiovascular Mortality**

ASA + Pooled Ticagrelor ASA + Rivaroxaban 2.5mg


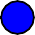

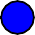

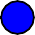

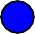

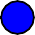


ASA

Clopidogrel 75mg

Rivaroxaban 5mg

##

## Supplementary figure 8: Forest Plot

**
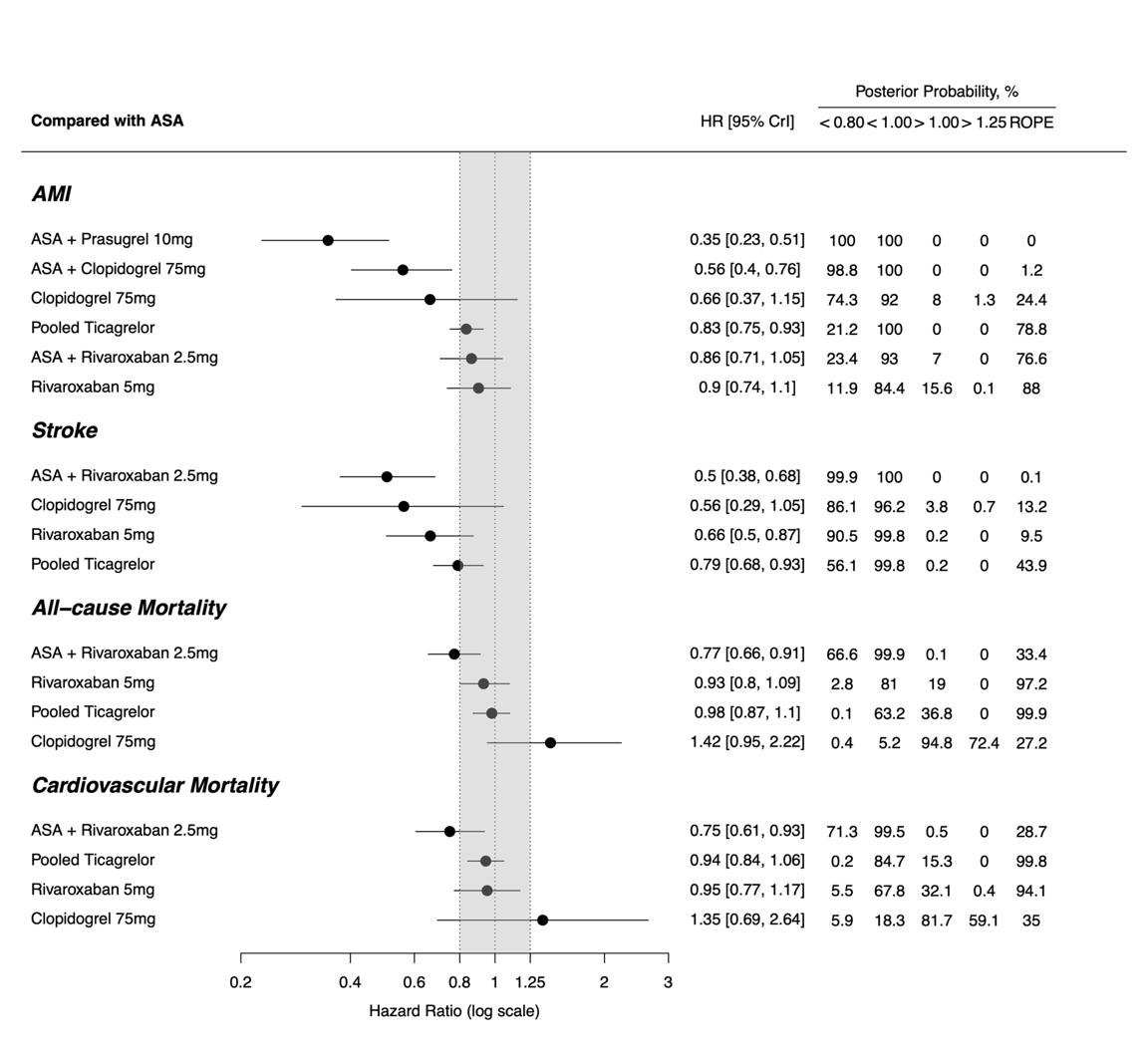
**

Treatment effects compared to ASA on MACE and Bleeding outcomes, ordered according to underlying SUCRA values. HR below 1.0 favors the experimental treatment. On the left, treatment names are depicted. In the middle, forest plot shows each treatment effect median and 95% credible intervals. Gray area corresponds to the ROPE (from 0.8 to 1.25 HR). On the right, exact effect sizes along with posterior probabilities are shown.

Abbreviations: ROPE, region of practical equivalence; HR, hazard ratio.

26

## League Tables

### Supplementary table 13-15: Median and 95% Credible Intervals

Acute Myocardial Infarction

| ASA 0.90 (0.74, 1.10) 0.66 (0.37, 1.15) 0.86 (0.71, 1.05) | 0.35 (0.23, 0.51) | 0.56 (0.40, 0.76) | 0.83 (0.75, 0.93) |
| --- | --- | --- | --- |
| Rivaroxaban 5mg 0.73 (0.40, 1.35) 0.96 (0.79, 1.17) | 0.38 (0.25, 0.60) | 0.62 (0.43, 0.90) | 0.93 (0.75, 1.16) |
| Clopidogrel 75mg 1.30 (0.73, 2.44) | 0.53 (0.26, 1.04) | 0.84 (0.45, 1.65) | 1.26 (0.71, 2.32) |
| ASA + Rivaroxaban 2.5mg 0.40 (0.26, 0.64) | | 0.65 (0.44, 0.94) | 0.97 (0.77, 1.20) |
| ASA + Prasugrel 10mg | | 1.60 (1.13, 2.21) | 2.40 (1.57, 3.59) |
|  | | ASA + Clopidogrel 75mg | 1.49 (1.07, 2.10) |
|  | |  | Pooled Ticagrelor |

Hazard ratios (95% credible interval) for the Acute Myocardial Infarction outcome. Treatments are shown in the diagonal. Comparisons between treatments should be read from left to right and the estimate is in the cell in common between the column- defining treatment and the row-defining treatment. A hazard ratio < 1.0 favors the column-defining treatment. For example, in the Rivaroxaban 5mg vs. ASA comparison, the corresponding HR (95% CrI) was 0.90 (0.74, 1.10), favoring Rivaroxaban 5mg (ie, HR < 1.0).

Ischemic Stroke

| ASA | 0.66 (0.50, 0.87) 0.56 (0.29, 1.05) 0.50 (0.38, 0.68) | 0.79 (0.68, 0.93) |
| --- | --- | --- |
|  | Rivaroxaban 5mg 0.84 (0.43, 1.67) 0.76 (0.56, 1.00) | 1.19 (0.87, 1.63) |
|  | Clopidogrel 75mg 0.90 (0.45, 1.80)  ASA + Rivaroxaban 2.5 | 1.41 (0.73, 2.68)  mg 1.57 (1.13, 2.20)  Pooled Ticagrelor |

Hazard ratios (95% credible interval) for the Ischemic Stroke outcome. Treatments are shown in the diagonal. Comparisons between treatments should be read from left to right and the estimate is in the cell in common between the column-defining treatment and the row-defining treatment. A hazard ratio < 1.0 favors the column-defining treatment. For example, in the Rivaroxaban 5mg vs. ASA comparison, the corresponding HR (95% CrI) was 0.66 (0.50, 0.87), favoring Rivaroxaban 5mg (ie, HR < 1.0).

All-cause and Cardiovascular Mortality

| ASA | 0.93 (0.80, 1.09) 1.42 (0.95, 2.22) | 0.77 (0.66, 0.91) | 0.98 (0.87, 1.10) |
| --- | --- | --- | --- |
| 0.95 (0.77, 1.17) | Rivaroxaban 5mg 1.52 (0.98, 2.41) | 0.83 (0.71, 0.98) | 1.05 (0.87, 1.28) |
| 1.35 (0.69, 2.64) | 1.42 (0.71, 2.82) Clopidogrel 75mg | 0.54 (0.35, 0.85) | 0.69 (0.44, 1.07) |
| 0.75 (0.61, 0.93) | 0.79 (0.63, 0.97) 0.56 (0.28, 1.12) | ASA + Rivaroxaban 2.5mg | 1.27 (1.03, 1.55) |
| 0.94 (0.84, 1.06) | 0.99 (0.79, 1.27) 0.70 (0.36, 1.37) | 1.26 (0.97, 1.60) | Pooled Ticagrelor |

Hazard ratios (95% credible interval) for the All-cause or Cardiovascular mortality outcomes. Treatments are shown in the diagonal. Results to the right of this diagonal (right upper half table) correspond to the All-cause mortality outcome. Results to the left (left lower half table) correspond to the Cardiovascular mortality outcome. Comparisons between treat- ments should be read from left to right and the estimate is in the cell in common between the column-defining treatment and the row-defining treatment. For both outcomes, a haz- ard ratio < 1.0 favors the column-defining treatment. For example, in the Rivaroxaban 5mg vs. ASA comparison for All-cause mortality, the corresponding HR (95% CrI) was 0.93 (0.80, 1.09), favoring Rivaroxaban 5mg (ie, HR < 1.0).

28

### Supplementary table 16-18: Posterior Probabilities of Hazard Ratio < 1.0

Acute Myocardial Infarction

| ASA 84.41 | 91.96 | 93.05 | 100.00 | 99.99 | 99.99 |
| --- | --- | --- | --- | --- | --- |
| Rivaroxaban 5mg | 84.44 | 66.90 | 100.00 | 99.49 | 75.36 |
|  | Clopidogrel 75mg | 19.30 | 96.45 | 69.55 | 21.73 |
|  |  | ASA + Rivaroxaban 2.5mg | 100.00 | 98.88 | 60.91 |
|  |  |  | ASA + Prasugrel 10mg | 0.34 | 0.00 |
|  |  |  |  | ASA + Clopidogrel 75mg | 0.84  Pooled Ticagrelor |

Posterior probabilities (%) of hazard ratio < 1.0 for the Acute Myocardial Infarction outcome. Treatments are shown in the diagonal. Comparisons between treatments should be read from left to right and the estimate is in the cell in common between the column-defining treatment and the row-defining treatment. A hazard ratio < 1.0 favors the column-defining treatment. For example, in the Rivaroxaban 5mg vs. ASA comparison, there was a posterior probability of 84.41% that the hazard ratio is below 1.0 (ie, there was a 84.41% probability for Rivaroxaban 5mg superiority).

Ischemic Stroke

| ASA 99.78 | 96.21 | 100.00 | 99.84 |
| --- | --- | --- | --- |
| Rivaroxaban 5mg 67.96 96.91 14.72 | | | |
| Clopidogrel 75mg | | 61.85 | 15.14 |
|  | | ASA + Rivaroxaban 2.5mg | 0.47 |
|  | |  | Pooled Ticagrelor |

Posterior probabilities (%) of hazard ratio < 1.0 for the Ischemic Stroke outcome. Treatments are shown in the diagonal. Comparisons between treatments should be read from left to right and the estimate is in the cell in common between the column-defining treatment and the row-defining treatment. A hazard ratio < 1.0 favors the column-defining treatment. For example, in the Rivaroxaban 5mg vs. ASA comparison, there was a posterior probability of 99.78% that the hazard ratio is below 1.0 (ie, there was a 99.78% probability for Rivaroxaban 5mg superiority).

All-cause and Cardiovascular Mortality

| ASA 81.00 | 5.22 | 99.88 | 63.21 |
| --- | --- | --- | --- |
| 67.85 Rivaroxaban 5mg 3.20 | | 99.01 | 31.13 |
| 18.30 15.59 | Clopidogrel 75mg | 99.55 | 95.09 |
| 99.51 98.32 | 95.00 | ASA + Rivaroxaban 2.5mg | 1.09 |
| 84.67 52.39 | 85.71 | 3.71 | Pooled Ticagrelor |

Posterior probabilities (%) of hazard ratio < 1.0 for the All-cause or Cardiovascular mortality outcomes. Treatments are shown in the diagonal. Results to the right of this diagonal (right upper half table) correspond to the All-cause mortality outcome. Results to the left (left lower half table) correspond to the Cardiovascular mortality outcome. Comparisons between treatments should be read from left to right and the estimate is in the cell in common between the column-defining treatment and the row-defining treatment. For both outcomes, a hazard ratio < 1.0 favors the column-defining treatment. For example, in the Rivaroxaban 5mg vs. ASA comparison for All-cause mortality, there was a posterior probability of 81.00% that the hazard ratio is below 1.0 (ie, there was a 81.00% probability for Rivaroxaban 5mg superiority).

30

### Supplementary table 19-21: Posterior Probabilities of Hazard Ratio < 0.8

Acute Myocardial Infarction

| ASA 11.95 | 74.31 | 23.38 | 100.00 | 98.76 | 21.18 |
| --- | --- | --- | --- | --- | --- |
| Rivaroxaban 5mg | 61.42 | 4.20 | 99.96 | 91.38 | 10.10 |
|  | Clopidogrel 75mg | 5.47 | 87.78 | 43.68 | 6.42 |
|  |  | ASA + Rivaroxaban 2.5mg | 99.92 | 86.95 | 4.42 |
|  |  |  | ASA + Prasugrel 10mg | 0.01 | 0.00 |
|  |  |  |  | ASA + Clopidogrel 75mg | 0.00  Pooled Ticagrelor |

Posterior probabilities (%) of hazard ratio < 0.80 for the Acute Myocardial Infarction outcome. Treatments are shown in the diagonal. Comparisons between treatments should be read from left to right and the estimate is in the cell in common between the column-defining treatment and the row-defining treatment. A hazard ratio < 1.0 favors the column-defining treatment. For example, in the Rivaroxaban 5mg vs. ASA comparison, there was a posterior probability of 11.95% that the hazard ratio is below 0.80.

Ischemic Stroke

ASA 90.46 86.09 99.90 56.12

Rivaroxaban 5mg 44.45 63.78 0.69

Clopidogrel 75mg 37.50 4.54

ASA + Rivaroxaban 2.5mg 0.00

Pooled Ticagrelor

Posterior probabilities (%) of hazard ratio < 0.80 for the Ischemic Stroke outcome. Treat- ments are shown in the diagonal. Comparisons between treatments should be read from left to right and the estimate is in the cell in common between the column-defining treatment and the row-defining treatment. A hazard ratio < 1.0 favors the column-defining treatment. For example, in the Rivaroxaban 5mg vs. ASA comparison, there was a posterior probability of 90.46% that the hazard ratio is below 0.80.

All-cause and Cardiovascular Mortality

| ASA | 2.84 | 0.35 | 66.61 | 0.09 |
| --- | --- | --- | --- | --- |
| 5.51 | Rivaroxaban 5mg | 0.26 | 33.65 | 0.43 |
| 5.90 | 5.30 | Clopidogrel 75mg | 95.43 | 74.46 |
| 71.29 | 54.85 | 85.20 | ASA + Rivaroxaban 2.5mg | 0.00 |
| 0.24 | 4.01 | 65.56 | 0.00 | Pooled Ticagrelor |

Posterior probabilities (%) of hazard ratio < 0.80 for the All-cause or Cardiovascular mor- tality outcomes. Treatments are shown in the diagonal. Results to the right of this diagonal (right upper half table) correspond to the All-cause mortality outcome. Results to the left (left lower half table) correspond to the Cardiovascular mortality outcome. Comparisons be- tween treatments should be read from left to right and the estimate is in the cell in common between the column-defining treatment and the row-defining treatment. For both outcomes, a hazard ratio < 1.0 favors the column-defining treatment. For example, in the Rivaroxaban 5mg vs. ASA comparison for All-cause mortality, there was a posterior probability of 2.84% that the hazard ratio is below 0.80.

32

### Supplementary table 22-24: Posterior Probabilities of Hazard Ratio > 1.25

Acute Myocardial Infarction

| ASA 0.06 | 1.32 | 0.00 | 0.00 | 0.00 | 0.00 |
| --- | --- | --- | --- | --- | --- |
| Rivaroxaban 5mg | 4.19 | 0.35 | 0.00 | 0.01 | 0.41 |
|  | Clopidogrel 75mg | 55.04 | 0.91 | 11.80 | 51.45 |
|  |  | ASA + Rivaroxaban 2.5mg | 0.00 | 0.04 | 1.20 |
|  |  |  | ASA + Prasugrel 10mg | 92.62 | 99.90 |
|  |  |  |  | ASA + Clopidogrel 75mg | 85.66  Pooled Ticagrelor |

Posterior probabilities (%) of hazard ratio > 1.25 for the Acute Myocardial Infarction outcome. Treatments are shown in the diagonal. Comparisons between treatments should be read from left to right and the estimate is in the cell in common between the column-defining treatment and the row-defining treatment. For both outcomes, a hazard ratio < 1.0 favors the column-defining treatment. For example, in the Rivaroxaban 5mg vs. ASA comparison, there was a posterior probability of 0.06% that the hazard ratio is above 1.25.

Ischemic Stroke

ASA 0.00 0.66 0.00 0.00

Rivaroxaban 5mg 13.48 0.00 38.48

Clopidogrel 75mg 18.57 64.20

ASA + Rivaroxaban 2.5mg 90.22

Pooled Ticagrelor

Posterior probabilities (%) of hazard ratio > 1.25 for the Ischemic Stroke outcome. Treat- ments are shown in the diagonal. Comparisons between treatments should be read from left to right and the estimate is in the cell in common between the column-defining treatment and the row-defining treatment. For both outcomes, a hazard ratio < 1.0 favors the column- defining treatment. For example, in the Rivaroxaban 5mg vs. ASA comparison, there was a posterior probability of 0.00% that the hazard ratio is above 1.25.

All-cause and Cardiovascular Mortality

| ASA | 0.00 | 72.41 | 0.00 | 0.00 |
| --- | --- | --- | --- | --- |
| 0.41 | Rivaroxaban 5mg | 80.41 | 0.00 | 3.99 |
| 59.11 | 64.08 | Clopidogrel 75mg | 0.00 | 0.41 |
| 0.00 | 0.00 | 1.29 | ASA + Rivaroxaban 2.5mg | 56.09 |
| 0.00 | 3.02 | 4.54 | 51.69 | Pooled Ticagrelor |

Posterior probabilities (%) of hazard ratio > 1.25 for the All-cause or Cardiovascular mor- tality outcomes. Treatments are shown in the diagonal. Results to the right of this diagonal (right upper half table) correspond to the All-cause mortality outcome. Results to the left (left lower half table) correspond to the Cardiovascular mortality outcome. Comparisons be- tween treatments should be read from left to right and the estimate is in the cell in common between the column-defining treatment and the row-defining treatment. For both outcomes, a hazard ratio < 1.0 favors the column-defining treatment. For example, in the Rivaroxaban 5mg vs. ASA comparison for MACE, there was a posterior probability of 0.00% that the hazard ratio is above 1.25.

34

### Supplementary table 25-27: Posterior Probabilities of Hazard Ratio within the ROPE

Acute Myocardial Infarction

| ASA 87.99 | 24.36 | 76.62 | 0.00 | 1.24 | 78.83 |
| --- | --- | --- | --- | --- | --- |
| Rivaroxaban 5mg | 34.39 | 95.45 | 0.04 | 8.61 | 89.49 |
|  | Clopidogrel 75mg | 39.49 | 11.31 | 44.52 | 42.12 |
|  |  | ASA + Rivaroxaban 2.5mg | 0.07 | 13.01 | 94.38 |
|  |  |  | ASA + Prasugrel 10mg | 7.36 | 0.10 |
|  |  |  |  | ASA + Clopidogrel 75mg | 14.34  Pooled Ticagrelor |

Posterior probabilities (%) of hazard ratio within the region of practical equivalence (ROPE), ie, between 0.80 and 1.25, for the Acute Myocardial Infarction outcome. Treatments are shown in the diagonal. Comparisons between treatments should be read from left to right and the estimate is in the cell in common between the column-defining treatment and the row-defining treatment. For both outcomes, a hazard ratio < 1.0 favors the column-defining treatment. For example, in the Rivaroxaban 5mg vs. ASA comparison, there was a posterior probability of 87.99% that the hazard ratio is within the ROPE.

Ischemic Stroke

| ASA 9.54 | 13.25 | 0.10 | 43.88 |
| --- | --- | --- | --- |
| Rivaroxaban 5mg 42.08 36.23 60.84 | | | |
| Clopidogrel 75mg | | 43.92 | 31.26 |
|  | | ASA + Rivaroxaban 2.5mg | 9.78 |
|  | |  | Pooled Ticagrelor |

Posterior probabilities (%) of hazard ratio within the region of practical equivalence (ROPE), ie, between 0.80 and 1.25, for the Ischemic Stroke outcome. Treatments are shown in the diagonal. Comparisons between treatments should be read from left to right and the esti- mate is in the cell in common between the column-defining treatment and the row-defining treatment. For both outcomes, a hazard ratio < 1.0 favors the column-defining treatment. For example, in the Rivaroxaban 5mg vs. ASA comparison, there was a posterior probability of 9.54% that the hazard ratio is within the ROPE.

All-cause and Cardiovascular Mortality

| ASA 97.16 | 27.24 | 33.39 | 99.91 |
| --- | --- | --- | --- |
| 67.85 Rivaroxaban 5mg 19.32 | | 66.35 | 95.59 |
| 18.30 15.59 | Clopidogrel 75mg | 4.58 | 25.12 |
| 99.51 98.32 | 95.00 | ASA + Rivaroxaban 2.5mg | 43.91 |
| 84.67 52.39 | 85.71 | 3.71 | Pooled Ticagrelor |

Posterior probabilities (%) of hazard ratio within the region of practical equivalence (ROPE), ie, between 0.80 and 1.25, for the All-cause or Cardiovascular mortality outcomes. Treat- ments are shown in the diagonal. Results to the right of this diagonal (right upper half table) correspond to the All-cause mortality outcome. Results to the left (left lower half ta- ble) correspond to the Cardiovascular mortality outcome. Comparisons between treatments should be read from left to right and the estimate is in the cell in common between the column-defining treatment and the row-defining treatment. For both outcomes, a hazard ratio < 1.0 favors the column-defining treatment. For example, in the Rivaroxaban 5mg vs. ASA comparison for All-cause mortality, there was a posterior probability of 97.16% that the hazard ratio is within the ROPE.

## Supplementary image 9: Ranks + SUCRA

##
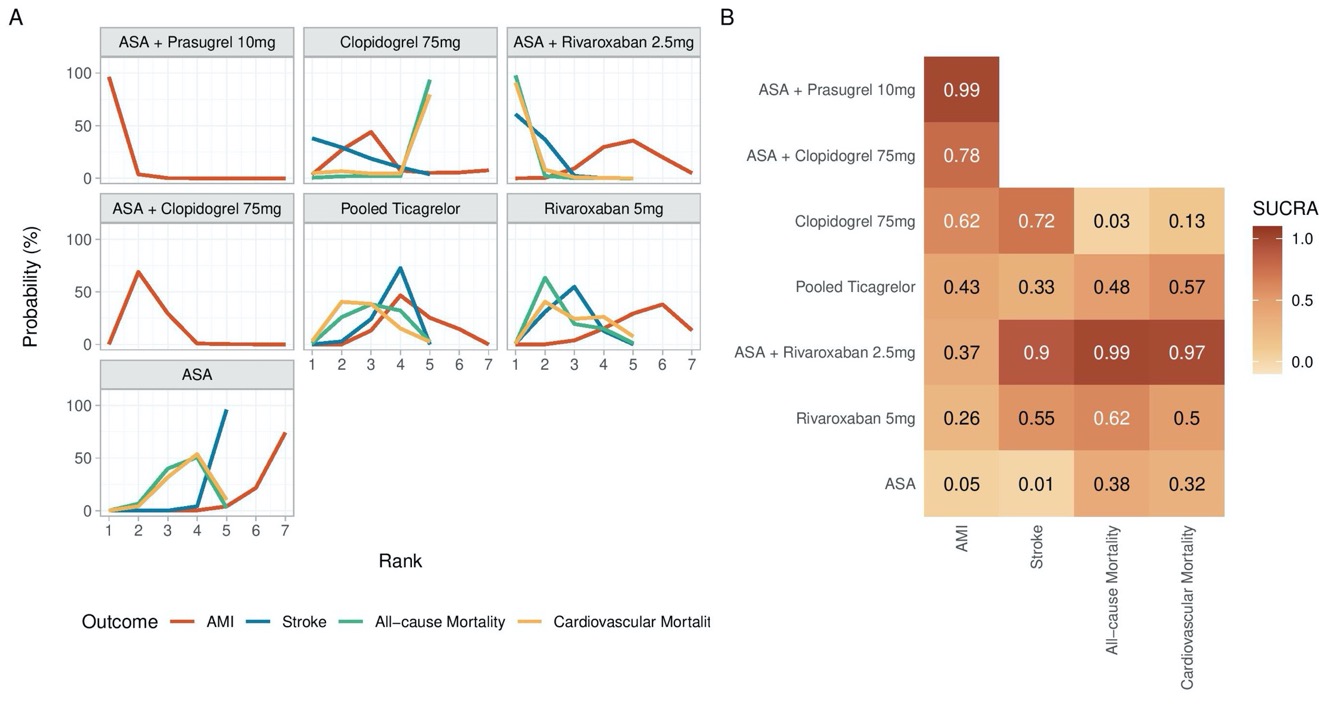


36

Panel A: Ranking probabilities for Acute Myocardical Infarction (AMI), Stroke, All-cause and Cardiovascular Mortality out- comes for each treatment. There are only AMI rankings for “ASA + Prasugrel 10mg” and “ASA + Clopidogrel 75mg” because there was no data available for other outcomes in corresponding studies. Panel B: Heatmap with corresponding SUCRA values. While each row corresponds to a treatment, each column depicts one outcome.

## Supplementary figure 10: Ranks with Uncertainty

**
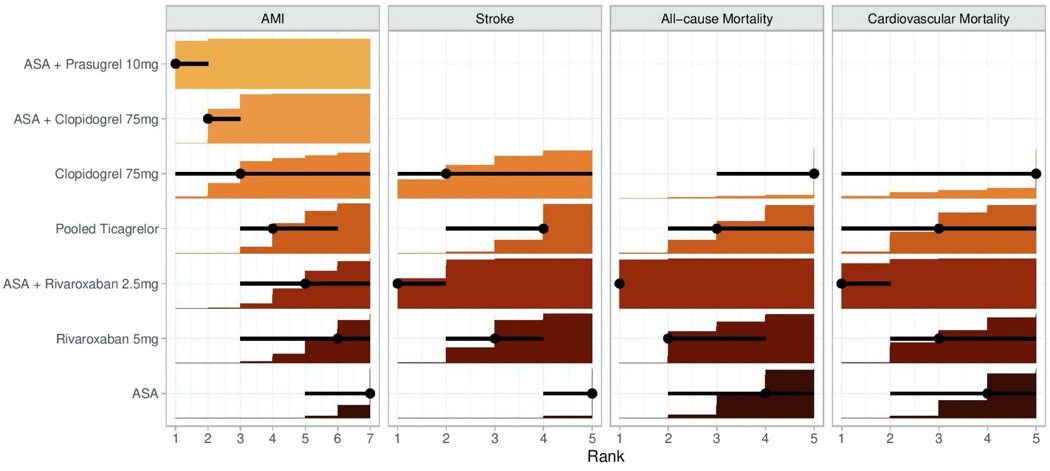
**

Marginal posterior distributions for the rank of each treatment. Each panel corresponds to a separate outcome, labeled on top. In each panel, there are a point estimate, interval bar, and bar plot for each treatment (Y-axis). The point estimate represents the median rank. The interval bar shows the 95% credible (quantile) interval of the underlying marginal posterior distribution. Lastly, the bar plot shows the cumulative distribution function (CDF).

37

# CINeMA

38

## Supplementary table 28-33: Pooled Ticagrelor Networks

### MACE


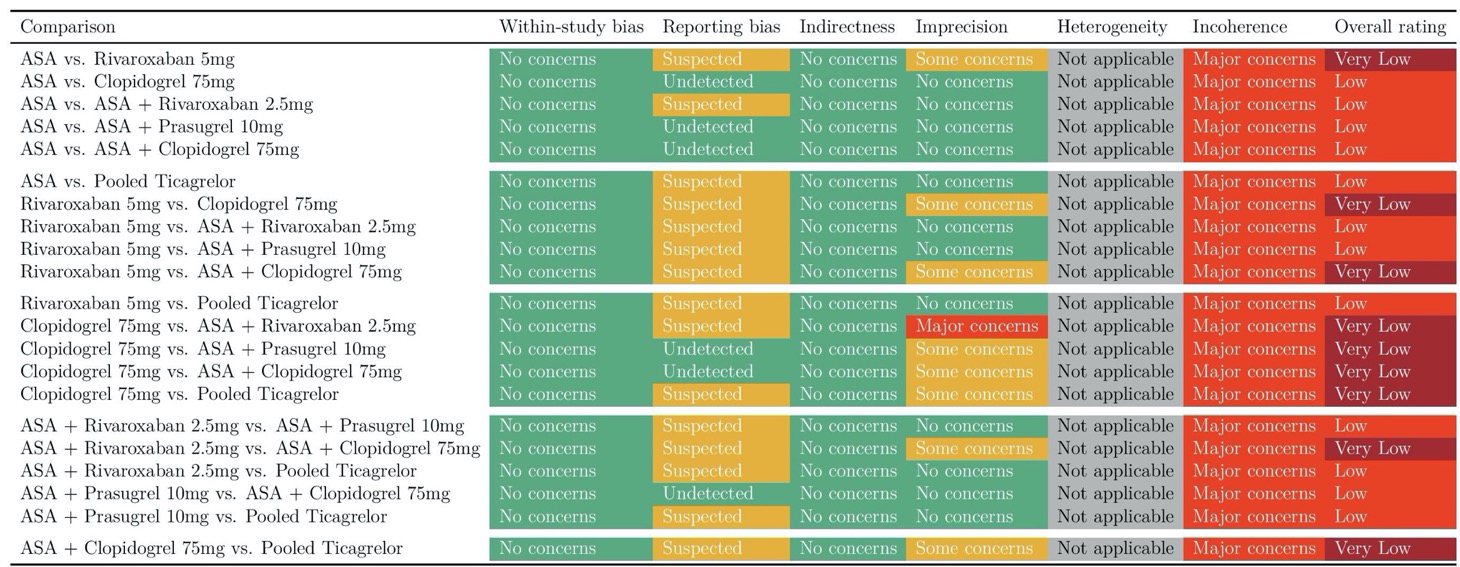


39

### Bleeding


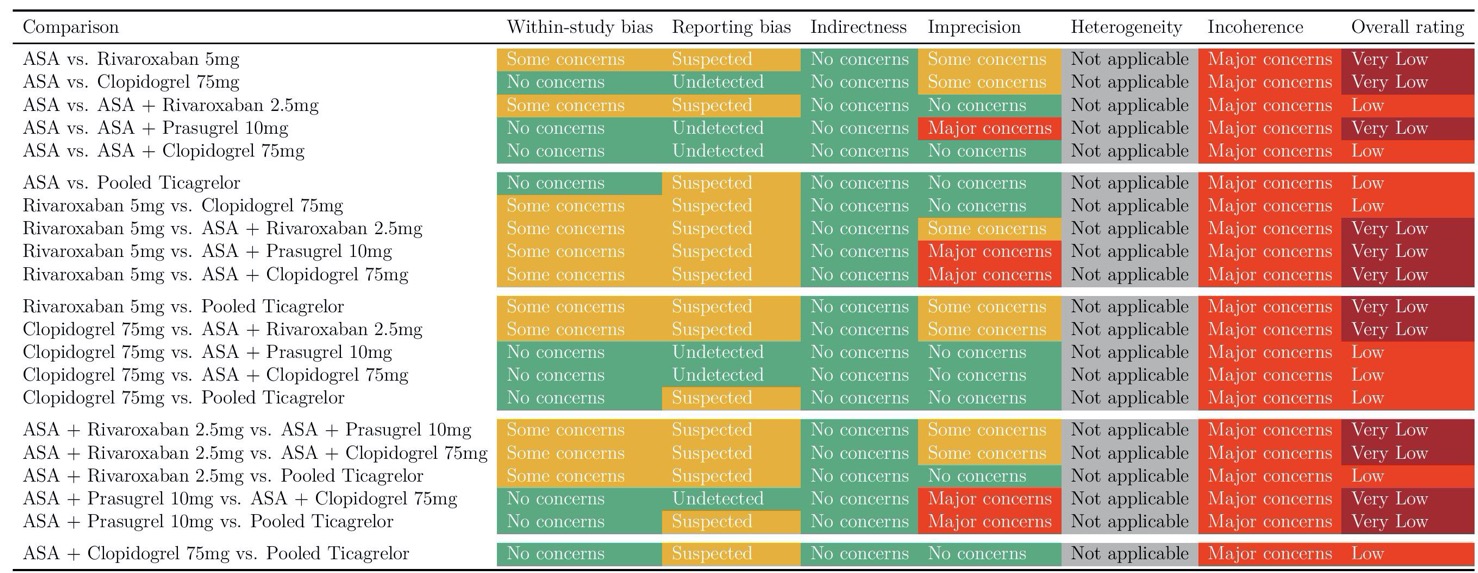


40

### Acute Myocardial Infarction

###
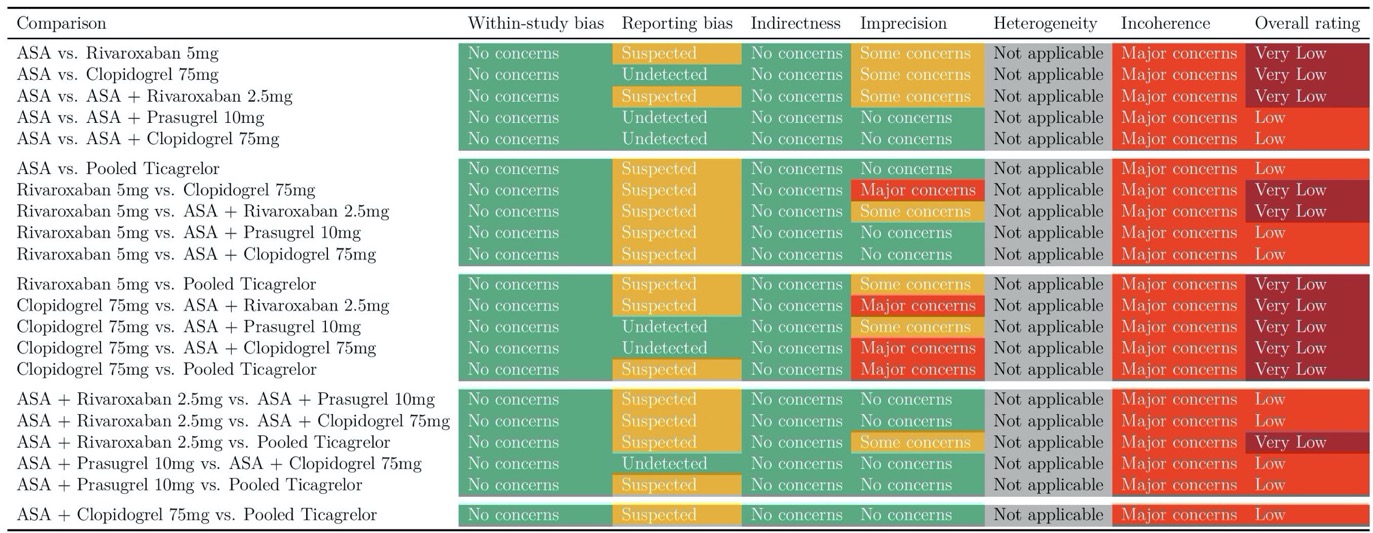


### Ischemic Stroke

###
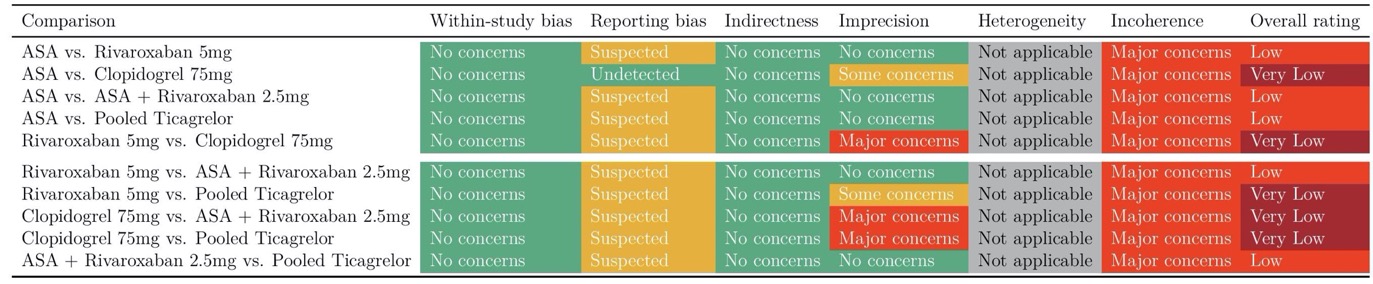


41

### All-cause Mortality


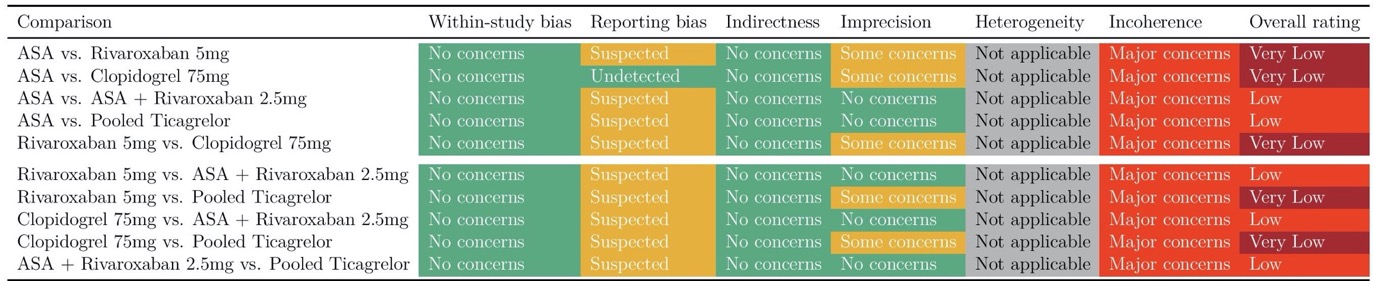


### Cardiovascular Mortality

###
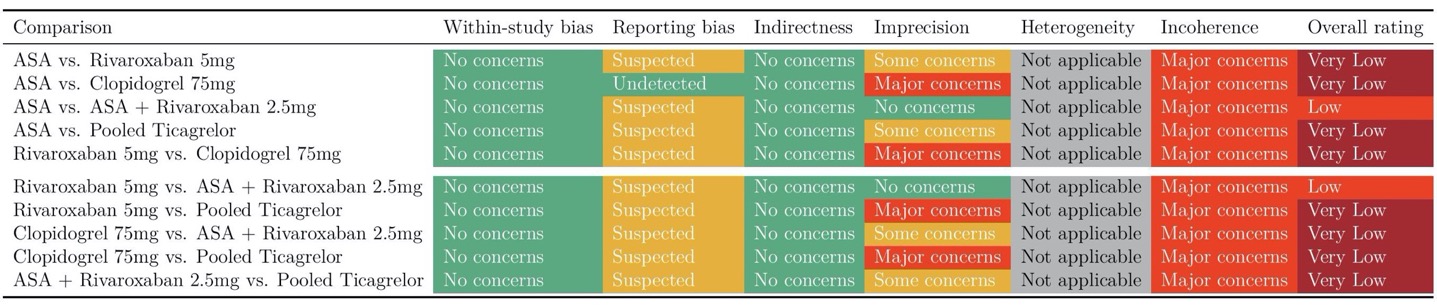


42

43

## Supplementary table 34-35: Separate Dosages Ticagrelor Networks

### MACE


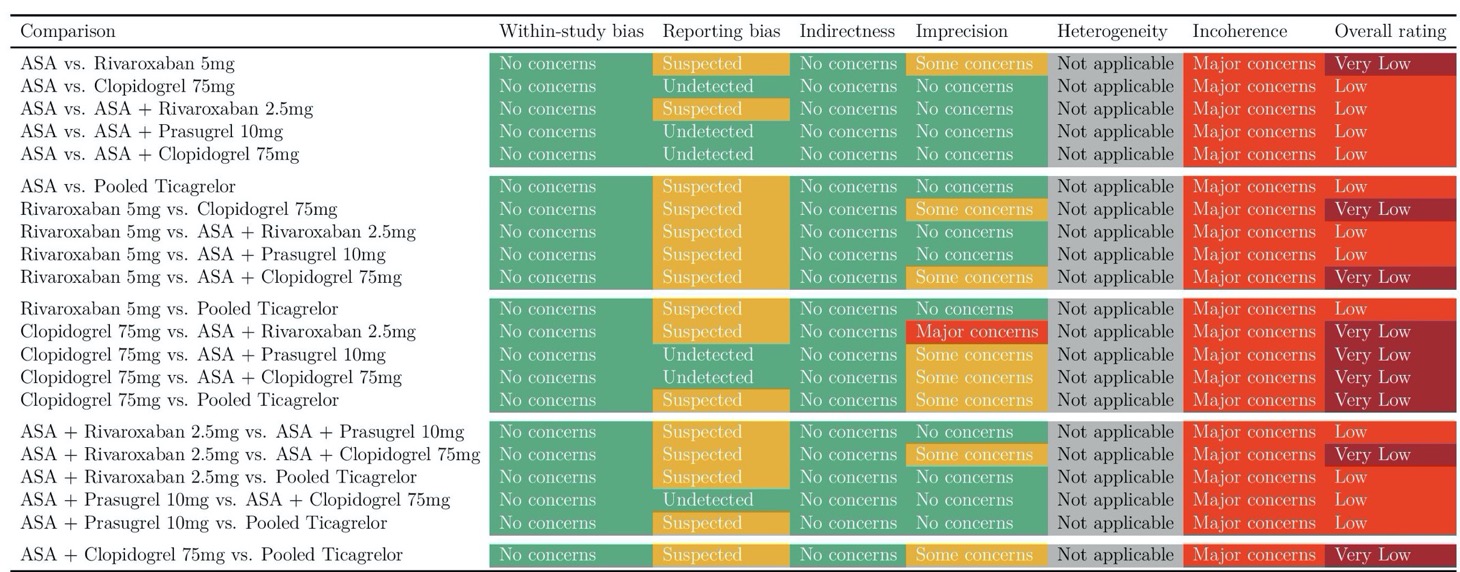


44

### Bleeding


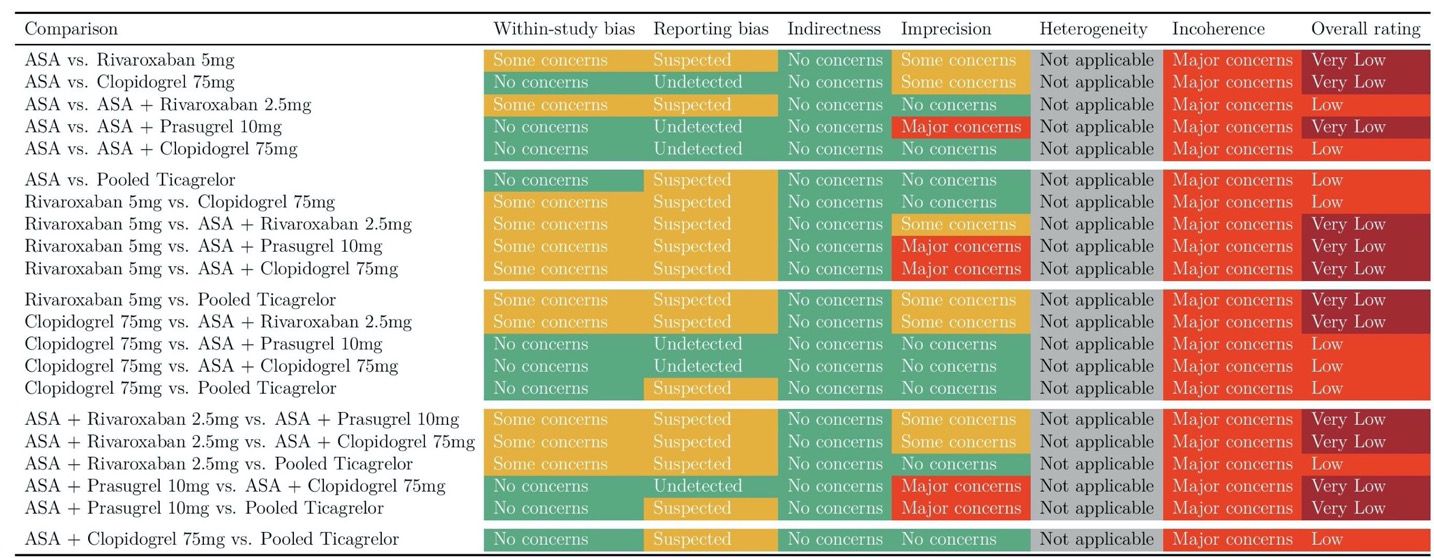

Supplement: Supplementary file 1 [file Datasheet1.docx]
